# AKAP6 and phospholamban colocalize and interact in HEK‐293T cells and primary murine cardiomyocytes

**DOI:** 10.14814/phy2.14144

**Published:** 2019-07-19

**Authors:** Farigol Hakem Zadeh, Allen C. T. Teng, Uros Kuzmanov, Paige J. Chambers, Allan R. Tupling, Anthony O. Gramolini

**Affiliations:** ^1^ Department of Physiology Faculty of Medicine University of Toronto Toronto Ontario; ^2^ Translational Biology and Engineering Program (TBEP) Ted Rogers Centre for Heart Research Toronto Ontario; ^3^ Department of Kinesiology University of Waterloo Waterloo Ontario

**Keywords:** A‐kinase anchoring protein (AKAP), Ca^2+^ transport, heart, protein kinase A (PKA), sarcoplasmic reticulum (SR)

## Abstract

Phospholamban (PLN) is an important Ca^2+^ modulator at the sarcoplasmic reticulum (SR) of striated muscles. It physically interacts and inhibits sarcoplasmic reticulum Ca^2+^ ATPase (SERCA2) function, whereas a protein kinase A (PKA)‐dependent phosphorylation at its serine 16 reverses the inhibition. The underlying mechanism of this post‐translational modification, however, remains not fully understood. Using publicly available databases, we identified A‐kinase anchoring protein 6 (AKAP6) as a candidate that might play some roles in PLN phosphorylation. Immunofluorescence showed colocalization between GFP‐AKAP6 and PLN in transfected HEK‐293T cells and cultured mouse neonatal cardiomyocytes (CMNCs). Co‐immunoprecipitation confirmed the functional interaction between AKAP6 and PLN in HEK‐293T and isolated adult rat cardiomyocytes in response to isoproterenol stimulation. Functionally, AKAP6 promoted Ca^2+^ uptake activity of SERCA1 in cotransfected HEK‐293T cells despite the presence of PLN. These results were further confirmed in adult rat cardiomyocytes. Immunofluorescence showed colocalization of both proteins around the perinuclear region, while protein–protein interaction was corroborated by immunoprecipitation of the nucleus‐enriched fraction of rat hearts. Our findings suggest AKAP6 as a novel interacting partner to PLN in HEK‐293T and murine cardiomyocytes.

## Introduction

In cardiomyocytes, SERCA2 is responsible for the majority of Ca^2+^ uptake from the cytosol into the SR (Li et al., 1998, Maclennan and Kranias 2003). SERCA2 activity is modulated via various biochemical modifications or protein interactions (Maclennan and Kranias 2003). PLN is a 52‐amino acid transmembrane protein that interacts and inhibits SERCA2 function at the SR (Simmerman and Jones, 1998, Maslennikov et al., 1995). During end‐stage heart failure, there is a decrease in SERCA2 activity due to a decrease in SERCA2 expression and an increased inhibitory effect of PLN on SERCA2 (Sankaranarayanan et al., 2016). Previous studies have identified three PLN mutations in heart failure patients with inherited cardiomyopathies. The first mutation is an Arg9 to Cys missense mutation (PLN‐R9C), leading to an increased affinity toward PKA (Schmitt et al. 2003). The second mutation creates a de novo premature stop codon at Leu 39 (PLN‐L39Stop). PLN‐L39Stop is barely detectable in human cardiac tissues and is misrouted to the plasma membrane in transfected HEK‐293 cells (Haghighi et al., 2003). The third PLN mutation has a deleted Arg14 residue (PLN‐RΔ14), disrupting the di‐arginine motif on positions 13 and 14 (R13/R14) of PLN that mediates retrograde transport of PLN protein with COPI vesicles from the Golgi apparatus back to the SR (Haghighi et al., 2006, Sharma et al., 2010). Loss of R14 residue also disrupts the PKA recognition sequence (R‐R‐X‐S/T) and PKA‐mediated phosphorylation, rendering PLN‐RΔ14 highly inhibitory toward SERCA2 (Haghighi et al., 2006).

Ca^2+^‐cycling is critical to cardiac physiology, and PLN plays a key role in modulating Ca^2+^ uptake via an inhibitory interaction with SERCA2 (Zhao et al., 2009). Phosphorylation of PLN on either residue Ser16 by PKA or Thr17 by Ca^2+^/calmodulin‐dependent protein kinase II (CamKII) reverses PLN's affinity toward SERCA2 (Tada and Katz 1982). During stress and sympathetic response in the heart, *β*‐adrenergic stimulation activates G protein‐coupled receptors and downstream adenylyl cyclase (AC) to generate cyclic AMP from ATP (Maclennan and Kranias 2003). Activated PKA regulates Ca^2+^‐cycling in cardiomyocytes by phosphorylating different proteins such as dihydropyridine receptor, ryanodine receptor (RyR), and PLN (Maclennan and Kranias 2003; Vassilatis et al., 2003).

Specificity of PKA toward its effectors is mediated by A‐kinase anchoring proteins (AKAPs) (Ruehr et al. 2004; Diviani et al. 2013). Seventeen different membranous AKAP genes have been identified in the human heart (Nauert et al. 1997; Kapiloff et al. 1999; Ruehr et al. 2004; Russell et al. 2006; Diviani et al. 2011, 2013; Smith et al. 2018).The activities of these AKAPs are not exclusive to PKA, and other enzymes such as phosphoprotein phosphatases (PPs) and phosphodiesterases (PDEs) may be recruited to AKAPs for modulating downstream singling pathways (Nauert et al. 1997; Eide et al. 1998; Fraser et al. 1998; Mizuno et al. 2001). A previous study showed that AKAP7 could mediate PKA‐dependent PLN phosphorylation, while disrupting PLN and AKAP7 interaction abolished phosphorylation of Ser16 on PLN in vitro (Lygren et al. 2007). However, AKAP7‐null and wild‐type mice did not differ in cardiac responses to *β*‐adrenergic receptor activation (Jones et al. 2012). These AKAP7‐null mice were generated by deleting the seventh exon of the gene, which may not be sufficient to ablate completely the expression of all AKAP isoforms (Smith et al. 2018). It is also feasible that other AKAPs could also mediate PLN phosphorylation in the heart. Therefore, we sought to search additional AKAP candidates that also coordinate PLN phosphorylation in the heart.

## Materials and Methods

### Bioinformatic analyses

Human RNA‐seq data from the Human Proteome Map (HPM), the Human Protein Atlas (HPA), the Genotype‐Tissue Expression (GTEx), the Functional Annotation of Mammalian Genome 5 (FANTOM5), and the Gene Expression Omnibus (GEO; Accession Number GSE89714) were downloaded (11 March 2017) and compiled for comparing the expression of AKAPs in human hearts (Edgar et al. 2002; Takahashi et al. 2012, Consortium, 2013; Uhlén et al. 2015). Tissue enrichment and localization data from HPA were used to establish the protein expression profile of AKAPs in the nondisease and disease human hearts.

AKAP phosphoproteomes in nonhypertrophic and hypertrophic cardiomyopathy (HCM) human hearts were analyzed using our previous data (available via ProteomeXchange; PXD011107) (Kuzmanov et al. 2018). Phosphosite levels were analyzed using quantile, width adjustment in Perseus software (Tyanova et al. 2016). The first, second, and third quartiles (q1, q2, q3) were calculated from the distribution of all values. The second quartile (the median) was subtracted from each value to center the distribution. Values were then divided by the width in an asymmetric way. All values that were positive following subtraction of the median were divided by q3‐q2, while all negative values were divided by q2‐q1.

### Cardiomyocyte isolation and cell culture

HEK‐293T cells were maintained and transfected as described previously (Ryan et al., 2011). Isoproterenol was used at a final concentration of 10 *μ*mol/L in cell culture media, for 10 min (Bachmann et al., 2016). After 10 min, isoproterenol was removed and cells were lysed and prepared for immunoprecipitation. All animal experiments were approved by the University of Toronto Animal Care and Use Committee. We generated and assessed neonatal cardiomyocyte cultures from CD1 mouse neonates as published previously (Chis et al., 2012). The isolation of adult rat ventricular cardiomyocyte was performed as previously described (Lee et al., 2018).

### Lentiviral and adenoviral expression studies

All plasmids used in this study were previously reported, including pLex‐mCherry‐Sec61*β*, pEGFP‐AKAP6, and pLex‐NF‐PLN (FLAG tag fused to the N terminal of PLN‐WT, RΔ14, S16A, R9C, and L39Stop) (Kapiloff et al. 1999; Teng et al., 2015, Haghighi et al., 2006, Kimura et al. 1996). Lentiviral packaging was performed in HEK‐293T cells as previously described, and virus was applied to isolated cardiomyocytes at 50 multiplicities of infection (MOI), which achieved >90% transduction efficiency (Teng et al., 2015). Adenoviral particles carrying short hairpin ribonucleic acid (shRNA) against rat AKAP6 or scrambled shRNA sequence were kindly provided by Hrishikesh Singh Thak and Michael Kapiloff (Pare et al. 2005a). Cardiomyocytes were transduced at 1000 MOI for 72 h.

### Immunoprecipitation and immunofluorescence

For immunoprecipitation, 500 *μ*g of rat cardiac lysate was incubated with 4 *μ*g of antibody overnight at 4°C. The next day, the protein A/G agarose beads (Pierce) were blocked with 5% BSA in IP buffer (20 mmol/L HEPES; pH 7.4, 150 mmol/L NaCl, 1% Triton X‐100, 5 mmol/L EDTA, 5% glycerol), supplemented with Roche complete protease inhibitor tablets (Roche, 11697498001) and phosphatase inhibitors for 1 h, and were added to the samples to be incubated for 2 h at 4°C. The samples were then washed and prepared for Western blotting.

Hearts from CD1 mice at 8–12 weeks of age were used for subcellular fractionation as described previously (Gramolini et al. 2007). Briefly, 100 mg of cardiac tissues in 1 mL sucrose lysis buffer (250 mmol/L sucrose, 50 mmol/L Tris‐HCl; pH7.4, 5 mmol/L MgCl_2_) was homogenized at 10,000 rpm with a handheld homogenizer (Polytron, PT1200E) for 1 min, incubated on ice for 30 min, and spun at 800 g for 15 min. The pellet containing a nuclear enriched fraction was dissolved in nuclear extraction buffer. Non‐nuclear impurity was removed by centrifuged at 80,000*g* for 35 min, and the nuclear fraction was resuspended in 50 mmol/L HEPES (pH 7.4), 10% glycerol, 1 mmol/L benzamidine, 1 mmol/L dithiothreitol (DTT), 5 mmol/L ethylenediaminetetraacetic acid (EDTA), 5 mmol/L ethylene glycol‐bis (*β*‐aminoethyl ether)‐N,N,N′,N′‐tetraacetic acid (EGTA), 25 mmol/L sodium fluoride (NaF), 40 mmol/L sodium glycerophosphate, and 1 mmol/L sodium pyrophosphate (Kapiloff et al. 2001). For immunoblots, protein lysates were extracted from transfected HEK‐293T cells in IP buffer, CMNCs in radioimmunoprecipitation assay (RIPA, 20 mmol/L Tris‐HCl; pH7.4, 150 mmol/L NaCl, 1% Triton X‐100, 0.5% sodium deoxycholate, 0.1% sodium dodecyl sulfate) buffer, and hearts also in RIPA buffer, supplemented with a protease inhibitor and phosphatase inhibitors. 30–100 *μ*g of total proteins was fractionated by SDS‐PAGE gels and transferred to 0.45 *μ*m nitrocellulose membranes (Bio‐Rad). Blots were incubated with the primary antibody overnight at 4°C and with the HRP‐conjugated secondary antibody for 1 h at 22°C. Finally, the ECL Western blotting substrate (Pierce) was applied to membranes for 5 min before detected by ChemiDoc imaging systems (Bio‐Rad). Primary antibodies and their dilution for immunoblotting are included below: Mouse monoclonal anti‐FLAG antibody (Abcam, 1:1000), 1D11 mouse monoclonal antibody against PLN (kind gift from Dr. R. Johnson, 1:5000), mouse monoclonal anti‐PLN antibody (Abcam, 1:1000), rabbit monoclonal A285 anti‐phosphorylated PLN antibody (kindly provided by Dr. R. Johnson, 1:1000), rabbit polyclonal anti‐AKAP6 antibody (EMD, Millipore Sigma, 1:1000), rabbit polyclonal anti‐PKA II*α* regulatory sc‐908 antibody (Santa Cruz, 1:1000), mouse monoclonal anti‐*α*‐tubulin antibody (Hybridoma, 1:1000), and mouse monoclonal anti‐lamin A/C sc‐7292 antibody (Santa Cruz, 1:1000).

For immunofluorescence, isolated murine cardiomyocytes were fixed by 4% paraformaldehyde in PBS, permeabilized and blocked in blocking solution (1×PBS, 3% fetal bovine serum, 0.1% Triton X‐100) for 10 min on ice. Samples were incubated overnight at 4°C with the primary antibodies diluted in blocking solution. Next morning, cells were washed and incubated with secondary antibodies and were imaged by ZEISS spinning disk confocal microscope. The primary antibodies and their dilution used for immunofluoresence include mouse monoclonal anti‐FLAG antibody (Abcam, 1:500), 2D12 mouse monoclonal anti‐PLN antibody (Abcam, 1:500), and mouse anti‐mAKAP Antibody anti‐mAKAP (OR017.720 – BioLegend, 1:500). The fluorophore‐conjugated secondary antibodies (Alexa Fluor 488 & Alexa Fluor 633, 1:500 Life Technologies) were diluted in blocking solution.

### SERCA activity measurements

ATPase activity of transfected HEK‐293T cells was measured using a spectrophotometric plate reader (SPECTRAMAX plus; Molecular Devices) across Ca^2+^ concentrations ranging from pCa 7.0 to 4, as previously described by Duhamel et al. (2007). Details are available in the Supplemental information.

### Statistical analysis

Using GraphPad Prism 5, one‐way ANOVA analysis was utilized to test the differences and the significance among more than two groups with Tukey's multiple‐comparison test as post hoc. To compare two groups, unpaired *t*‐test was performed. The data are expressed as mean ± SEM. A *P*‐value <0.05 was considered significant.

More details on the experimental procedures are available on “Supporting information: Material and Methods” section of the Supporting Information [https://doi.org/10.6084/m9.figshare.7597517 or https://figshare.com/s/e2f3685b0c62d59378b7] document.

## Results

### RNA expression profile of all AKAPs in human heart tissues

**Figure 1:**
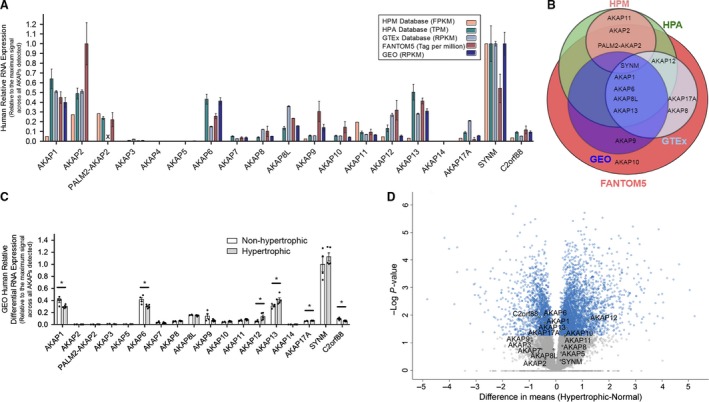
RNA expression analyses of AKAP6 in human hearts. (A) RNA‐seq analyses of human AKAP candidates in heart tissues using HPM (*n* = 85, individuals, unit: fragments per kilobase million (FPKM), HPA (*n* = 4, heart tissues, unit: transcripts per kilobase million (TPM), GTEx (*n* = 412, heart tissues, unit: reads per kilobase million (RPKM), FANTOM5 (*n* = 4, individuals, unit: tags per million), and GEO (*n* = 4, individuals, unit: reads per kilobase million (RPKM) database. An “X” is incorporated where RNA‐seq data are not found. The data show the mean ± SEM expressions normalized to the maximum mean signal all across within each dataset. (B) Venn diagram summary of AKAPs with high RNA expression level (with the relative detection threshold of 0.1). (C) RNA‐seq analysis of human AKAP candidates in nonhypertrophic and hypertrophic adult human heart using GEO data, relative to the highest expression across all samples. These results are shown as mean  ± SEM; *n* = 4 for nonhypertrophic and *n* = 5 for hypertrophic samples. *P < 0.05 comparing non‐hypertrophic versus hypertrophic patients’ RNA expression in the heart for each AKAP. (D) Volcano plot of the whole RNA‐seq dataset from GEO. Log‐transformed p values associated with individual proteins plotted against difference in expression levels between nonhypertrophic and hypertrophic human hearts. *P < 0.05, blue scatterplots. The AKAPs aliases/other names, normalized mean and SEM of RNA‐seq data for AKAPs of nonhypertrophic and hypertrophic, and patients were included in Table S1–S3 within the Supporting Information [https://doi.org/10.6084/m9.figshare.7597517 or https://figshare.com/s/e2f3685b0c62d59378b7] document.

A previous study showed that AKAP7 could bridge between PKA and PLN in vitro (Rigatti et al. 2015), but targeted AKAP7 deletion in mouse hearts did not affect PLN phosphorylation following the adrenergic receptor activation (Jones et al. 2012), suggesting other cardiac AKAPs may be functionally compensating for the loss of AKAP7. As adaptor molecules, the levels of AKAP proteins mostly account for their functions. Thus, we first screened several transcriptomic, proteomic, and phosphoproteomic databases of human cardiac tissues at nondisease versus disease states. RNA levels of AKAPs were compared with publicly available RNA‐sequencing (RNA‐seq) data from the Human Protein Atlas (HPA), the Human Proteome Map (HPM), the gene‐level dataset and Gene Expression Omnibus repository (GEO; Accession Number GSE89714), Genotype‐Tissue Expression (GTEx), and the Functional Annotation of Mammalian Genome 5 (FANTOM5) (Fig. 1A) (Edgar et al. 2002; Takahashi et al. 2012, Consortium, G 2013; Kim et al. 2014; Uhlén et al. 2015). Although AKAP3 and AKAP4 are not expressed in the heart, in this study, they were used as negative controls with close to 0 detection level. Furthermore, PALM2‐AKAP2 was not detected in GTEx data. In the HPM, HPA, GTEx, and GEO databases, the mean RNA expression levels of the AKAPs were normalized to the expression levels of SYNM which had the highest expression while in FANTOM5 database, the expression levels were normalized to AKAP2 levels (Fig. 1A). Transcripts of AKAP1, AKAP6, AKAP8L, and AKAP13 were consistently expressed at a higher level compared to remaining AKAPs in all datasets (Fig. 1A and B). Next, we sought to determine whether these transcript levels differ in the human disease model of hypertrophic cardiomyopathy, because impaired Ca^2+^ uptake is a common factor in failing hearts. Figure 1C showed that the transcripts of AKAP1, AKAP6, AKAP12, AKAP13, AKAP17A, and C2orf88 (chromosome 2 open‐reading frame 88) were different between nondisease versus disease conditions. To visualize AKAPs with fold changes versus statistical significance (*P* < 0.05), a volcano plot was used (Kuzmanov et al. 2018). Figure 1D shows 7 AKAPs had a significant alteration in transcript levels between human nonhypertrophic and hypertrophic samples, including AKAP1, AKAP6, AKAP10, AKAP12, AKAP13, AKAP17A, and C2orf88.

### Protein expression profile of human AKAPs and protein localization

**Figure 2:**
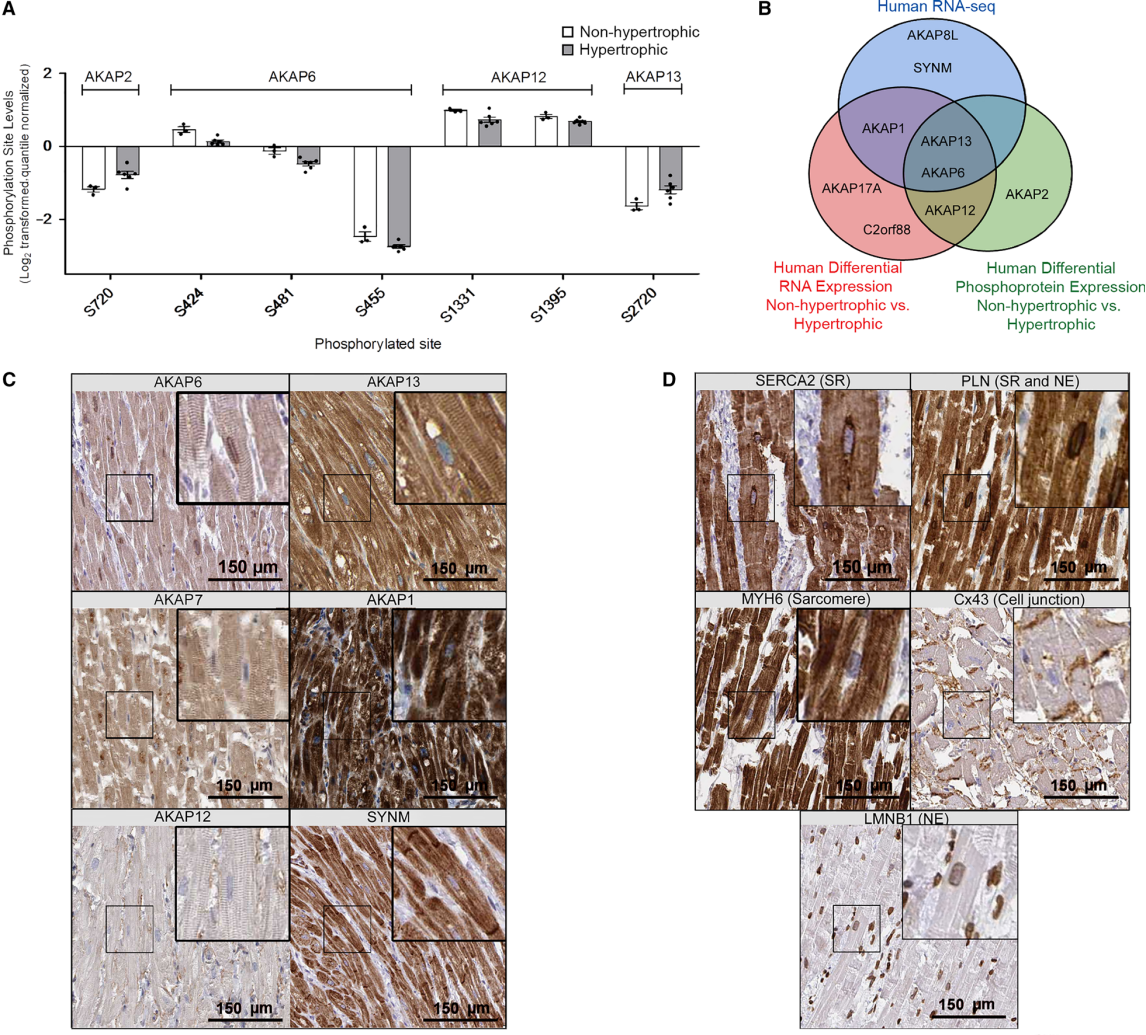
Phosphoproteomic analyses of AKAP6 in hypertrophic versus nonhypertrophic human hearts. (A) Significantly different phosphorylation sites of AKAP candidates (Log2 transformed, quantile‐normalized) from human nonhypertrophic versus hypertrophic cardiac tissues. (B) Venn diagram depicting the overlap of all AKAPs with high RNA‐seq, differential RNA, and differential phosphoprotein detect levels. (C) Representative immunohistochemistry of adult human heart sections. Data were obtained from the Human Protein Atlas (HPA) database (https://www.proteinatlas.org/). Scale bar, 150 *μ*m. (D) Representative staining of control proteins (sarco(endo)plasmic reticulum ATPase, SERCA2; phospholamban, PLN; myosin heavy chain 6, MYH6; connexin 43, Cx43; lamin B1 receptor, LMNB1). Scale bar, 150 *μ*m.

In order to identify AKAPs that have alterations in their phosphorylation and functionality during disease states, we investigated previous phosphoproteomic data collected from human HCM patients (Data available via ProteomeXchange; identifier PXD011107). Phosphosite levels were analyzed using quantile, width adjustment in Perseus software (Tyanova et al. 2016). The first, second, and third quartiles (q1, q2, q3) were calculated from the distribution of all values. The second quartile (the median) was subtracted from each value to center the distribution. Values were then divided by the width in an asymmetric way. All values that were positive following subtraction of the median were divided by q3‐q2, while all negative values were divided by q2‐q1. Differences in the detected phosphorylation sites among AKAPs were plotted (Fig. 2A). We identified phosphorylation sites for four major AKAPs and that AKAP2 (S720), AKAP6 (S424, S481 and S455), AKAP12 (S1331 and S1395), and AKAP13 (S2720) were differentially identified between the hypertrophic versus unaffected human hearts (*P* < 0.05). The only two AKAPs whose expression (Fig. 1C, RNA‐seq) and phosphorylation status (Fig. 2A, phosphoproteomics) were significantly altered in failing human hearts were AKAP6 and AKAP13 (Fig. 2B).

We next examined subcellular localization of AKAP1, AKAP6, AKAP7, AKAP12, AKAP13, and SYNM in the human heart available in the Human Protein Atlas (Uhlén et al. 2015). Figure 2C shows that AKAP6 was positive on the SR and the nuclear membrane (NE), while AKAP13 staining was predominantly cytosolic. By contrast, AKAP1, AKAP12, and SYNM had minimal to no sarcomeric or SR staining pattern, with no apparent nuclear envelope staining (Fig. 2C). As controls, staining was also compared to abundant cardiac markers, including SERCA2, PLN, myosin heavy chain 6 (MYH6), connexin 43 (Cx43), and lamin B1 (LMNB1) (Fig. 2D). Together, based on these results, it is suggested that AKAP6 is a potential candidate for facilitating PLN phosphorylation in the heart.

### AKAP6 localization in HEK‐293T cells

**Figure 3:**
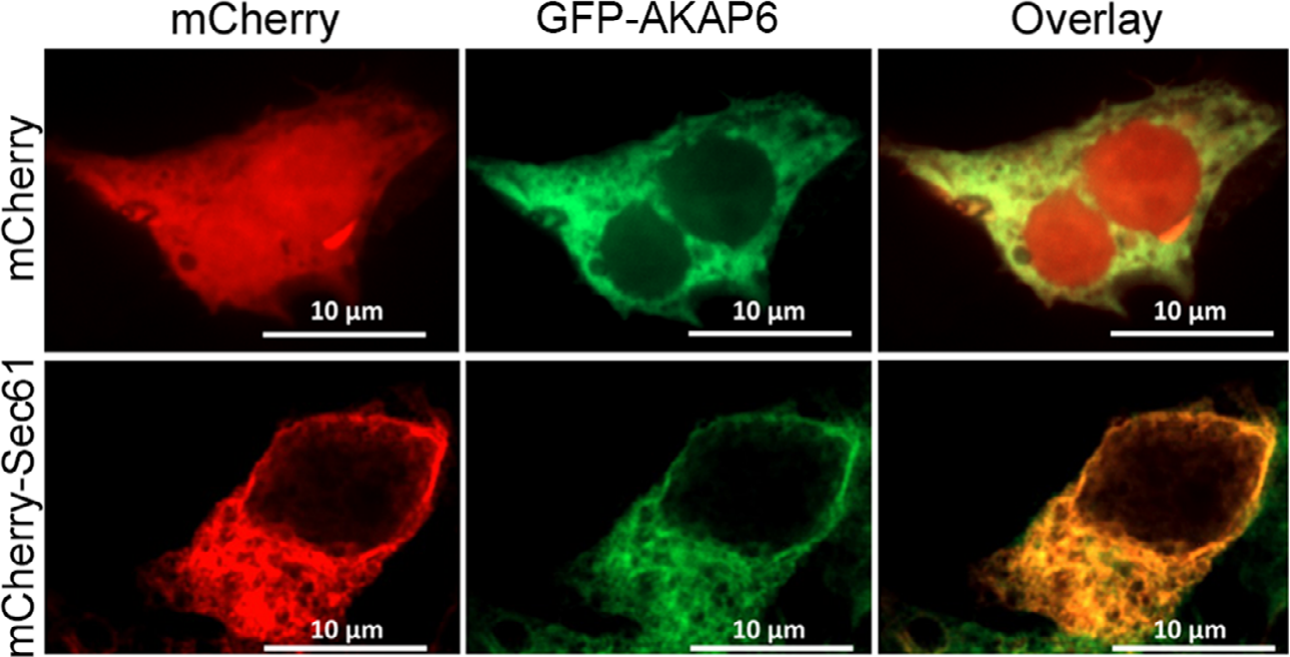
GFP‐AKAP6 localized to the endoplasmic reticulum in HEK‐293T cells. Cells were transfected with GFP‐AKAP6 and mCherry as a control or mCherry‐Sec61 Following 48 h post‐transfection, cells were imaged by confocal microscopy using the 633 nm wavelength channel (632 nm excitation channel, 647 nm emission channel; for mCherry), demonstrated in red; 488 nm channel (493 nm excitation channel, 517 nm emission channel; for GFP‐AKAP6), shown in green. 18 ± 1% of GFP‐AKAP6 was colocalized with 27 ± 1% of mCherry (Pearson's coefficient = 0.3 ± 0.05). 83 ± 1% of GFP‐AKAP6 was colocalized with 91 ± 1% mCherry‐Sec61 in HEK‐293T cells (Pearson's coefficient = 0.91 ± 0.01; *n* = 3 independent transfection experiments, at least 8 cells were included per calculation. *N* = 25 cells total). Scale bar, 10 *μ*m.

Next, we sought to determine whether AKAP6 would localize to the endoplasmic reticulum (ER)/SR region, starting in HEK‐293T cells. Immunofluorescence shows that GFP‐tagged AKAP6 (GFP‐AKAP6) colocalized with an endoplasmic reticulum marker mCherry‐Sec61 (Fig. 3), whereas mCherry control did not. Specifically, three‐dimensional reconstructions of these cells using Imaris showed 83 ± 1% of GFP‐AKAP6 signals colocalized with 91 ± 1% mCherry‐Sec61, with a Pearson's coefficient of 0.91 ± 0.01 (25 cells were quantified) (Fig. 3), while, in the negative control condition, 18 ± 3% of GFP‐AKAP6 was colocalized with 27 ± 1% of mCherry (Pearson's coefficient = 0.3 ± 0.05). GFP‐AKAP6 was also found in the perinuclear region of transfected HEK‐293T cells. Together, these results corroborated the findings of AKAP6 localized to the SR and the nuclear envelope (NE) in HEK‐293T cells.

### AKAP6 and PLN colocalize and co‐immunoprecipitate in HEK‐293T cells

**Figure 4:**
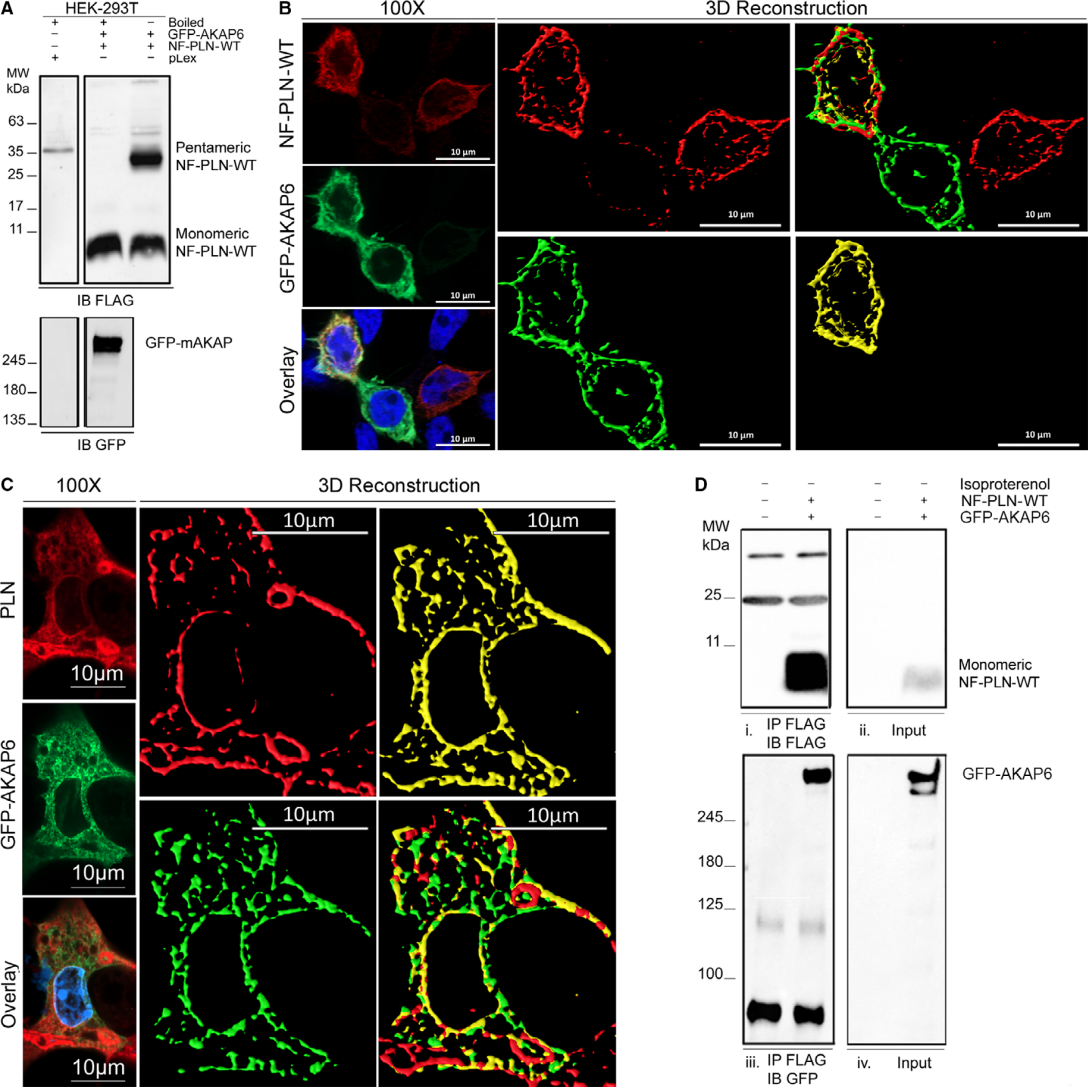
AKAP6 interaction with NF‐PLN‐WT in HEK‐293T cells. (A) Immunoblots confirmed overexpression of NF‐PLN and GFP‐AKAP6 in transfected HEK‐293T cells. Same blots split and processed with the same intensity scaling. Top blotted with anti‐FLAG antibody to detect NF‐PLN: First lane: control empty pLex transfection after 5 min boiling. Second lane: NF‐PLN and GFP‐AKAP6 transfection with 5 min boiling. Third lane: NF‐PLN and GFP‐AKAP6 transfection after no boiling. Bottom blotted with anti‐GFP antibody to detect GFP‐AKAP6: for GFP‐AKAP6. First lane: control empty pLex transfection after 5 min boiling. Second lane: NF‐PLN and GFP‐AKAP6 transfection with 5 min boiling. (B) Confocal imaging and 3D reconstruction showing colocalization of GFP‐AKAP6 and NF‐PLN (WT) in HEK‐293T cells (*n* = 3 independent transfection experiments, at least 10 cells were included per calculation, *N* = 30 total cells). Scale bar, 10 *μ*m. (C) Immunofluorescence showing colocalization between PLN and GFP‐AKAP6 signals in neonatal mouse cardiomyocyte cultures (*n* = 3 independent transfection experiments, at least 6 cells were included per calculation, *N* = 20 total cells). Immunofluorescence was performed with the following: (1) 1D11 (anti‐PLN) antibody for PLN detection, (2) anti‐GFP antibody for GFP‐AKAP6 detection. Scale bar, 10 *μ*m. (D) Immunoprecipitation showing NF‐PLN (WT) and GFP‐AKAP6 physically interacted in transfected HEK‐293T cells. All samples were boiled prior to electrophoresis. (1) NF‐PLN pull‐down with anti‐FLAG antibody and detection of NF‐PLN through immunoblotting with anti‐FLAG antibody. The bands observed at 25 kDa and 50 kDa are IgG the light chain and heavy chain. (2) NF‐PLN Lysate input signal detection through immunoblotting with anti‐FLAG antibody. (3) NF‐PLN pull‐down with anti‐FLAG antibody and detection of GFP‐AKAP6 through immunoblotting with anti‐GFP antibody. The bands observed close to 100 kDa are IgG aggregates formed by boiling prior to electrophoresis. (4) GFP‐AKAP6 Lysate input signal detection through immunoblotting with anti‐GFP antibody. Full immunoblots of all the blots were included in Figure S1 within the Supporting Information [DOI: 0.6084/m9.figshare.7597517 or https://figshare.com/s/e2f3685b0c62d59378b7] document.

We next explored whether AKAP6 and PLN would colocalize in transfected HEK‐293T cells. Immunoblots first confirmed successful expression and detection of GFP‐AKAP6 and FLAG‐tagged PLN (NF‐PLN) in transfected cells (Fig. 4A). Immunofluorescence and three‐dimensional reconstructions of 30 independently transfected cells showed 58 ± 2% GFP‐AKAP6 colocalized with 77 ± 3% NF‐PLN with a mean Pearson's coefficient of 0.69 ± 0.02 (Fig. 4B). GFP‐AKAP6 and endogenous PLN also colocalized in 30 independently examined CMNCs (Fig. 4C). To determine whether GFP‐AKAP6 and PLN biochemically interact, co‐immunoprecipitation (Co‐IP) of NF‐PLN and GFP‐AKAP6 was performed with transfected HEK‐293T lysates. Figure 4D shows NF‐PLN immunoprecipitation led to GFP‐AKAP6 co‐precipitation. We were unable to immunoprecipitate AKAP6 with either the Biolegend or Millipore antibody, and thus, reverse Co‐IPs were not possible. Together, our results show AKAP6 and PLN could colocalize and biochemically interact in HEK‐293T cells.

### SERCA activity profile upon AKAP6 and PLN overexpression in HEK‐293T

**Figure 5:**
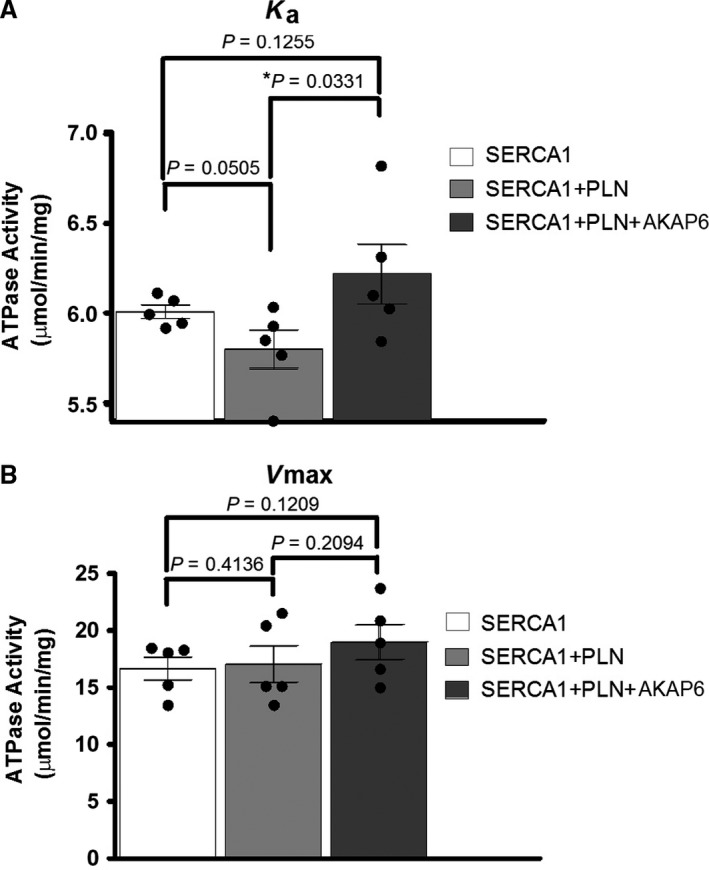
AKAP6 overexpression relieved PLN inhibition of SERCA1 in HEK‐293T cells. Ca^2+^ ATPase assays showing (A) *K*_a_ of SERCA1 and (B) *V*_max_ of SERCA1 in HEK‐293T cells transfected with SERCA1, SERCA1 + PLN, SERCA1 + PLN+AKAP6. These results are presented as mean  ± SD; *n* = 5. *P < 0.05. AKAP6 overexpression did not affect *V*_max_ of SERCA1, but increased *K*_a_ of SERCA1, indicating higher Ca^2+^ affinity of SERCA1 in HEK‐293T cells.

We next sought to determine the functional importance of PLN and AKAP6 interaction with Ca^2+^ ATPase assays that would compare the effect of AKAP6‐PLN‐SERCA1, PLN‐SERCA1, and SERCA1 only in transfected HEK‐293T cells. In this experiment, the Ca^2+^ affinity (K_a_) of SERCA1 slightly (albeit insignificantly) dropped when PLN (WT) was cotransfected (from 6.0 ± 0.1 _p_Ca units in SERCA1 only to 5.8 ± 0.2 _p_Ca units in SERCA1 + PLN, *P* = 0.05). Interestingly, upon the overexpression of AKAP6, the K_a_ significantly shifted to 6.2 ± 0.4 _p_Ca units compared to the SERCA1 + PLN group (Fig. 5A, *P = 0.03). All assays showed similar *V*_max_ (SERCA1, 16.7 ± 2.3 *μ*mol/min/mg, SERCA1 + PLN, 17.1 ± 3.6 *μ*mol/min/mg; Fig. 5B, *n* = 5, *P* > 0.1). Our results show that the presence of AKAP6 could reverse the inhibitory effect of PLN on SERCA1 in HEK‐293T cells.

### AKAP6 and PLN interactions

**Figure 6:**
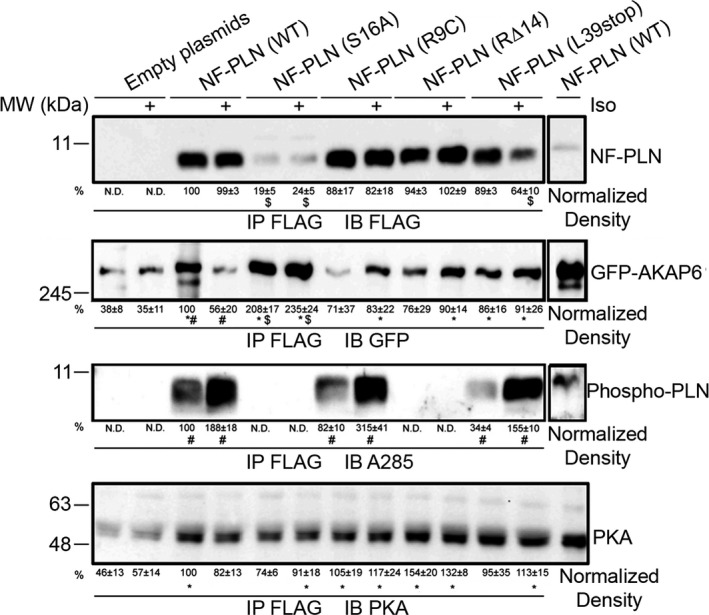
AKAP6 and PLN mutant interactions following isoproterenol stimulation of HEK‐293T cells. Co‐IP was performed using anti‐Flag antibody to immunoprecipitate NF‐PLN (WT, S16A, R9C, RΔ14, L39Stop). Samples were immunoblotted with anti‐FLAG antibody for NF‐PLN variants, anti‐GFP antibody for GFP‐AKAP6, A285 antibody for phospho‐PLN, and anti‐PKA antibody for PKA. Densitometry was performed for all bands, and the adjusted volume was normalized to the amount of protein immunoprecipitated with NF‐PLN (WT). These results are expressed in percentage as mean  ± SEM; *n* = 3. *P < 0.05 versus NF‐PLN. ^$^ *P* < 0.05 versus PLN‐WT. ^#^ *P* < 0.05, control versus isoproterenol treatment. Full immunoprecipitation blots of NF‐PLN, GFP‐AKAP6, phospho‐PLN, and PKA (*n* = 3 experiments) are available in the Supporting Information [https://doi.org/10.6084/m9.figshare.7597517 or https://figshare.com/s/e2f3685b0c62d59378b7] document.

To explore any physical interaction between AKAP6 and PLN mutants (S16A, R9C, RΔ14, or L39Stop), Co‐IP experiments were performed in transfected HEK‐293T cells, because no commercially available AKAP6 antibody could be used for immunoprecipitation. Figure 6 shows that all NF‐LN constructs were immunoprecipitated with FLAG antibody, but the level of NF‐PLN (S16A) precipitated was much less than NF‐PLN (WT) conditions (19 ± 5% in untreated NF‐PLN (S16A) compared to 100% in untreated NF‐PLN (WT); 24 ± 5% for isoproterenol‐treated NF‐PLN (S16A) compared to 99 ± 3% for isoproterenol‐treated NF‐PLN (WT)). Consistent with Figure 4D, NF‐PLN (WT) and GFP‐AKAP6 formed a biochemical interaction in HEK‐293T cells. Isoproterenol treatment, which promoted PLN phosphorylation at the Ser16 residue, however, reduced PLN‐AKAP6 interaction (56 ± 20% in isoproterenol‐treated versus 100% in untreated samples, *P* < 0.05, Fig. 6). Interestingly, three disease‐causing PLN mutants (R9C, RΔ14, and L39Stop) had increased biochemical interactions with AKAP6 in response to isoproterenol stimulation. Finally, the NF‐PLN (S16A) had the strongest interaction (208 ± 17%) with AKAP6 of all tested PLN variants. In addition, isoproterenol treatments did not change this interaction (Fig. 6). Phosphorylation status of PLN proteins was also assessed by immunoblots. Upon isoproterenol stimulation, three PLN (WT, R9C, and L39Stop) had increased phosphorylation signals, while the phosphorylation status on two other PLN (RΔ14 and S16A) was not detectable (Fig. 6). Lastly and as expected, in all cases, the PKA was detected in the co‐precipitated protein complexes, because PKA was possibly anchored to the precipitated AKAP6. Nevertheless, NF‐PLN (RΔ14) showed higher, albeit not statistically significant, levels of PKA interaction (normalized density of 154 ± 20% untreated and 132 ± 8% isoproterenol‐treated) compared to the WT NF‐PLN 100% untreated and 82 ± 13% isoproterenol‐treated (Fig. 6). Together, our results show that PLN and variants biochemically interacted with AKAP6 differentially in HEK‐293T cells.

### Colocalization and biochemical interaction between PLN and AKAP6 in rat adult ventricular cardiomyocytes

**Figure 7:**
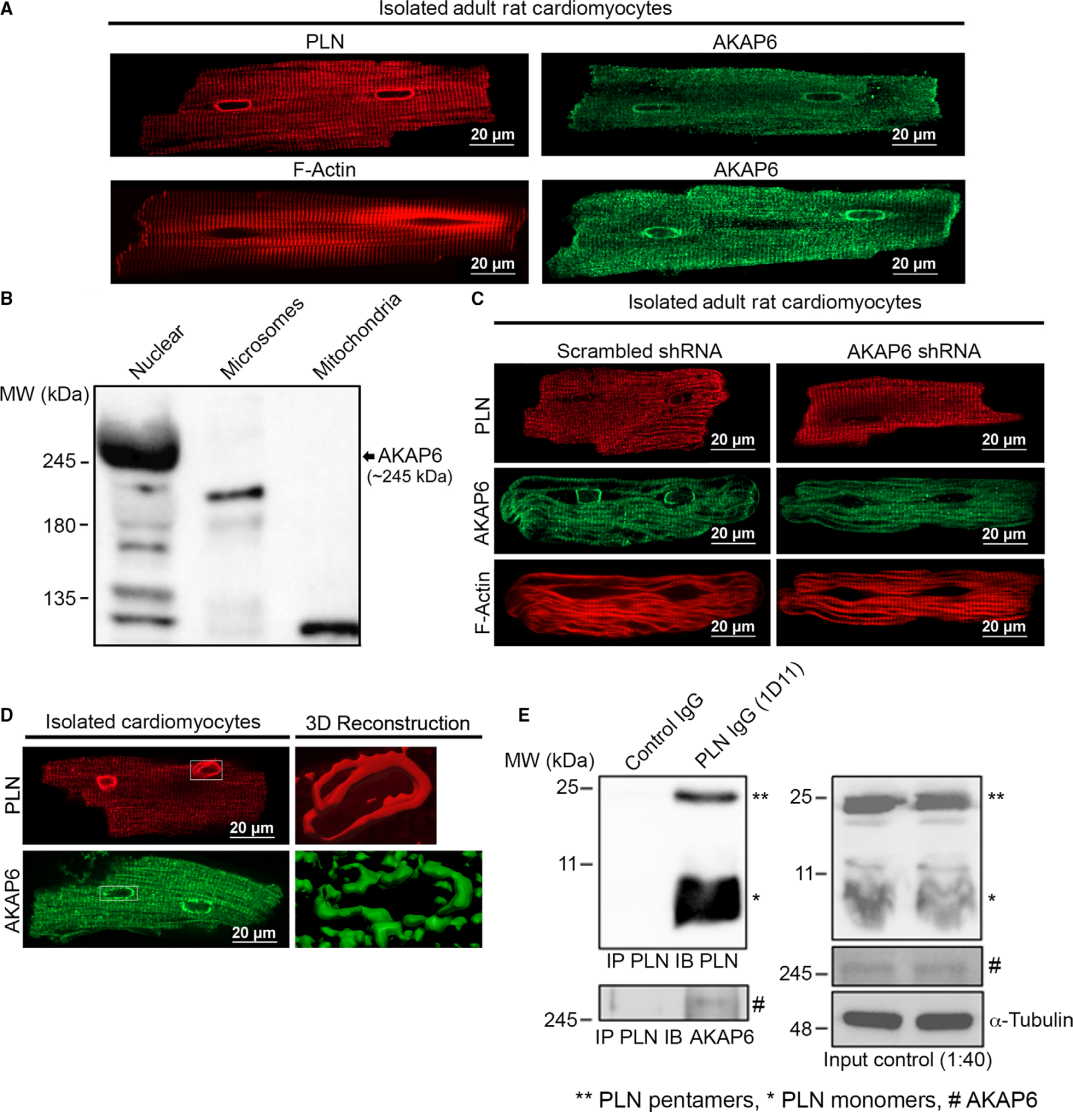
AKAP6 and PLN localization and immunoprecipitation in AVC. (A) (1) methanol fixation or (2) paraformaldehyde fixation followed by AKAP6, PLN, and phalloidin staining. No adenoviral transduction. Harvested 1 day after plating. Scale bar, 20 *μ*m. (B) AKAP6 subcellular localization in adult rat cardiomyocytes. (C) Immunofluorescence showing AKAP6 localization to the perinuclear region in adult rat cardiomyocytes. Adenovirus carrying scrambled (scram) or AKAP6 shRNA was used to silence endogenous AKAP6. AKAP6 shRNA resulted in the loss of protein signal around the perinuclear region. Harvested 3 days post‐transduction. Scale bar, 20 *μ*m. (D) 3D reconstruction of PLN and AKAP6 at the perinuclear, nuclear, and nuclear invagination in AVC (*n* = 45–50 cells per condition). No adenoviral transduction. Harvested 3 day post‐transduction. (E) Endogenous AKAP6 and PLN immunoprecipitation assays in adult rat hearts. IP was performed using 1D11 (anti‐PLN) antibody to immunoprecipitate endogenous PLN. Samples were immunoblotted with: 1D11 (anti‐PLN) antibody for PLN band detection, anti‐AKAP6 antibody for endogenous AKAP6 detection, PLN input, AKAP6 input, and *α*‐tubulin control input (*n* = 3). Full immunoblots of all the blots were included within the Supporting information [https://doi.org/10.6084/m9.figshare.7597517 or https://figshare.com/s/e2f3685b0c62d59378b7] document. Quantification of the number of WT (no transduction, *n* = 86 nuclei), Control (Scram) (*n* = 76 nuclei) and AKAP6 KD (*n* = 80 nuclei) cells containing or lacking AKAP6 staining are included in Table 1.

To investigate the colocalization of endogenous AKAP6 and PLN in the heart, we performed immunofluorescence in isolated rat adult ventricular cardiomyocytes (AVC). Figure 7A shows that endogenous AKAP6 and PLN independently localized to the SR and NE regions (Fig. 7A, top panels). Colocalization study was not possible because both AKAP6 and PLN antibodies were raised from mice. To determine the specific localization of endogenous AKAP6 in the heart, we performed a differential centrifugation, organellar fractionation of the whole heart to isolate a nuclear‐, microsomal/SR‐, and mitochondrial‐enriched fractions (Gramolini et al. 2007). Immunoblots for AKAP6 in these fractions showed that AKAP6 was highly expressed in the nuclear fraction, with no detection of the high molecular mass product in the other two fractions. A smaller product (220 kDa), however, was detected in the SR/microsomal fraction, which may represent a slicing AKAP6 variant (Fig. 7B) (Wu et al. 2010).

Since the biochemistry showed that the majority of the AKAP6 signal was in a nuclei‐enriched fraction, not the microsomal fraction that would contain the majority of the ER/SR, and the prevailing expression pattern was outside of the nucleus, we sought to carefully examine the specificity of the AKAP6 antibody by fluorescent microscopy. Rat adult cardiomyocytes were transduced with adenovirus carrying AKAP6 shRNA (Ad‐U6 AKAP6 7210D mp2A) or a scrambled shRNA (AKAP6 7210D mut.C), followed by immunofluorescence. We did not observe any differences in cells treated with the scrambled sequence (Fig. 7C). However, the AKAP6 knockdown cells lost the nuclear staining, while the striated SR staining pattern persisted. Quantification of these experiments in 45 AKAP6 shRNA cells showed that > 70% of them had no remaining nuclear staining, despite considerable fluorescence remaining at the sarcomere. More than 90% of the control cells (transfected with AKAP6 scrambled) showed prominent nuclear staining (Table 1). Together, these results show that most of the SR staining in the cardiomyocytes was due to nonspecific binding of the antibody, whereas the perinuclear staining was antibody‐specific. Focusing only on this specific perinuclear staining of AKAP6 and PLN, following 3D reconstruction of stained cardiomyocytes, both AKAP6 and PLN had distinct perinuclear staining (Fig. 7D).

**Table 1:** Quantification of the number of AVCs with nuclear stain upon transduction with adenovirus carrying scrambled or AKAP6 shRNA. (A) Quantification of the number of WT (no transduction, *n* = 86 nuclei), control (Scram) (*n* = 76 nuclei), and AKAP6 KD (*n* = 80 nuclei) cells containing or lacking AKAP6 staining. (B) Quantification of the number of WT (no transduction, *n* = 86 nuclei), control (Scram) (*n* = 76 nuclei), and AKAP6 KD (*n* = 80 nuclei) nuclei containing or lacking AKAP6 staining

Conditions		WT	Control	AKAP6‐KD
A				
Total cells		50	44	45
# Cells (% cells)	With nuclear stain	44 (88%)	40 (91%)	12 (26.7%)
B				
Total nuclei		86	76	80
#Nuclei (% nuclei)	With nuclear stain	77 (89.5%)	69 (90.8%)	20 (25%)

To determine the binding interaction between endogenous AKAP6 and PLN in the perinuclear and nuclear region of adult hearts, we performed Co‐IP of the endogenous PLN and AKAP6 with enriched nuclear fractions isolated from adult rat hearts. As demonstrated in Figure 7E, PLN precipitation in this fraction resulted in AKAP6 co‐precipitation compared to control samples showing no PLN and AKAP6 binding to nonspecific IgG. These data suggest that endogenous AKAP6 and PLN interact in the perinuclear region of adult rat cardiomyocytes.

## Discussion

One downstream effect of *β*‐adrenergic stimulation in the heart is the activation of the cAMP/PKA signaling pathway (Xiang and Kobilka 2003). Upon activation, PKA initiates the phosphorylation of several Ca^2+^‐cycling‐related proteins such as PLN and RyR2 (Reddy 1976; Tada and Katz 1982; Koss and Kranias 1996). AKAPs are important scaffold proteins that target PKA to its effectors in an organelle‐specific manner. The structural role of these AKAPs is not exclusive to PKA phosphorylation pathways, and they mediate the activity of several other enzymes at different subcellular regions (Nauert et al. 1997; Eide et al. 1998; Fraser et al. 1998; Mizuno et al. 2001). Thus, a comprehensive study of AKAP protein and their phosphorylation status in given cells or organs is important for mapping AKAP‐dependent signaling pathways, similar to conventional kinase‐mediated singling network. Here, we took the liberty of establishing the first AKAP transcriptome and phosphoproteome of human hypertrophic hearts (Figs. 1 and 2). To our best knowledge, this is the first systematic approach in profiling human cardiac AKAPs, although Scholten et al. 2007 reported the first mouse ventricular AKAP profiling. Human patient samples were limited to only hypertrophic cardiomyopathy due to limited availability of robust datasets, but the expansion of a similar analysis would likely extend the implications of our work. Another limitation includes human nonhypertrophic tissues, which were naturally difficult to obtain. Our study revealed that AKAP1, AKAP6, AKAP10, AKAP12, AKAP13, AKAP17A, and C2orf88 are differentially expressed following cardiac hypertrophy (Fig. 1D), while AKAP2, AKAP6, AKAP12, and AKAP13 had significantly altered phosphorylation status (Fig. 2A). Our findings were further validated by existing reports. For example, Li et al. (2017b) showed that targeted deletion of AKAP12 improved cardiac contraction, which was attributed to alteration in RYR2 phosphorylation. Evidently, Pare et al. (2005a) showed that AKAP6 mediated cardiac hypertrophy in rat hearts also via RYR2 phosphorylation. Collectively, these reports and our study illustrate the complexity and duplicated functions of AKAPs and thus the importance of profiling AKAP expression, post‐translational modifications, and protein–protein interactions in biological sources. Although proteogenomic (proteomic + genomic) approaches are often used for hypothesis generating, these tools are not without weaknesses. One potential limitation includes quantitative bias. For instance, signals of low expression proteins can be lost in a noisy background in proteomic searches. The signal‐to‐noise ratio can be further improved in prospective proteogenomic research with the advent of new technologies and computer algorithms.

PLN functions as an inhibitor to SERCA in striated muscle, but its inhibitory effect is reversed by PKA‐mediated phosphorylation on the Ser16 residue (Maclennan and Kranias 2003). Loss of PKA recognition site, as exemplified by the PLN‐RΔ14 mutant, renders it super inhibitory and is associated with arrhythmogenic right ventricular cardiomyopathy in human patients (van der Zwaag et al. 2012; Te Rijdt et al. 2019). It is currently under debate which AKAPs mediate PKA‐PLN interactions in cardiomyocytes. Previous studies identified AKAP 7/18δ as the mediator of PLN phosphorylation through PKA (Lygren et al. 2007). Experiments using the KO model of AKAP 7/18δ, nevertheless, did not show a significant disruption in cardiac function following adrenergic stimulation (Jones et al. 2012). Furthermore, a recent study illustrated a significant interaction between PLN and AKAP6. Yet, a conditional KO of AKAP5 in mice only partial perturbed adrenergic responses of the heart (Li et al. 2017a). Similarly, another recent study in mouse SYNM knockouts showed a very minor decrease in PKA‐RII phosphorylation with no concomitant changes in Ca^2+^‐transient kinetics (García‐Pelagio et al. 2018). In this study, we identified AKAP6 as a candidate that mediates PLN phosphorylation. Figures 5 and 7G show that PLN and AKAP6 biochemically interacted in transfected HEK‐293T cells and rat hearts. Immunofluorescence showed that PLN and AKAP6 independently colocalize at the perinuclear region of rat ventricular myocytes (Fig. 7F). Functionally, AKAP6 expression removed the inhibitory effect of PLN on SERCA1 (Fig. 6B). Together, this study suggests AKAP6 as a modulator for PLN phosphorylation at the perinuclear region of cardiomyocytes and, together with other AKAPs, co‐regulate PLN phosphorylation and function.

Our immunofluorescence studies showed that AKAP6 was localized to the ER/SR in transfected HEK‐293T cells and CMNCs, but was restricted to the perinuclear region in adult cardiomyocytes. Two hypotheses may account for this difference. First, there are considerable numbers of AKAP6 transcriptional or splicing variations. Some of those variants show SR staining patterns (Yang et al. 1998; Schulze et al. 2003; Faul et al. 2007), and others are restricted to the perinuclear region of myocytes (Kapiloff et al. 1999, 2001; Pare et al. 2005b; Kritzer et al. 2014). Our data using shRNA studies to highlight AKAP6 antibody‐specific staining, along with our organellar fractionation data, support preferential expression at the perinuclear domain in myocytes (Passariello et al. 2015). However, our finding could not rule out the possibility of the SR‐localized AKAP6 in adult rat cardiomyocytes, since autofluorescence from cardiac sarcomeric structure tends to overshadow the genuine, but weaker, immunofluorescent signals (Larcher et al. 2018). Secondly, a skewed ratio of endogenous Nuclear envelope spectrin‐repeat proteins 1 (Nesprin‐1) to the overexpressed AKAP6 may account for this discrepancy. Nesprin family proteins are intermediate filament proteins located on the outer nuclear membrane of cells. Currently, four Nesprin family members are identified, and they are responsible for connecting the nuclei to different types of cytoskeletons. While they all physically connect to the C‐terminal end of trimeric Sad1p‐UNC84 (SUN) complexes at the inner nuclear membrane, Nesprin 1/2 interact with F‐actin, Nesprin 3 with Plectin, and Nesprin 4 with microtubules in the cytoplasm (Rajgor and Shanahan 2013). A previous study showed that AKAP6 anchoring to the outer nuclear membrane depended on Nesprin 1 in African green monkey kidney COS7 cells (Pare et al. 2005b). In HEK‐293T cells, overexpressed AKAP6 may not be anchored to the outer nuclear membrane due partially to limited numbers of endogenous Nesprin 1 (Fig. 3). This observation reaffirms our finding that PLN and AKAP6 could interact at the Nesprin 1‐positive outer nuclear membrane of adult rat cardiomyocytes (Fig. 7F).

Our data showed an interaction between endogenous AKAP6 and PLN in the nuclear fraction of the adult rat hearts. Interestingly, AKAP6 is known to regulate PKA proteins activated through *β*2‐adrenergic receptors (AR) rather than *β*1‐AR at the level of the nucleus (Bedioune et al. 2018). It is known that unlike *β*1‐AR, *β*2‐AR is less involved in eliciting excitation–contraction events in cardiomyocytes (Xiao and Lakatta 1993). However, the high localization and interaction of PLN with AKAP6 at the perinuclear region suggests that, similar to RYR2 and *β*2‐AR activated PKA, PLN activity is regulated by AKAP6 at the level of the nucleus (Kapiloff et al. 2001). Interestingly, Zhou et al. 2015 showed that palmitoylated PLN was identified at the perinuclear region of adult mouse cardiomyocytes, while perinuclear PLN was suggested to play a role in modulating Ca^2+^ uptake and gene expression in adult mouse cardiomyocytes (Ljubojevic and Bers 2015; Wu et al. 2016; Chen et al. 2018). One future approach to elucidate the specific AKAP6 and PLN downstream effects can be investigated through knocking out AKAP6 and recording Ca^2+^‐cycling changes particularly at the nuclear envelope.

The Ca^2+^ uptake assays of transfected HEK‐293T cells with overexpressed SERCA1, SERCA1 + NF‐PLN, SERCA1 + NF‐PLN+GFP‐AKAP6 showed that while PLN expression inhibited SERCA1 activity by lowering the Ka of SERCA1, AKAP6 reversed PLN inhibition. These data suggest AKAP6 is capable of mediating PLN activation–inactivation processes, at least in HEK‐293T cells. One small caveat of using NF‐PLN is the unknown biochemical impact of negatively charged FLAG peptide to the full‐length PLN. Kimura et al. (1996) showed that FLAG tag, when directly fused to the membrane domain (PLN^28‐52^) of PLN, rendered it more inhibitory towards SERCA2. Similarly, Kleinschmidt et al. (1997) showed that negatively charged lipid had an inhibitory effect toward SERCA2. Conversely, phosphorylation of Ser16 or Thr17 on PLN reverses it binding toward SERCA2, despite the negative charges from phosphorylated residues. Future studies in how FLAG tag may impact PLN/AKAP6 interactions should be explored. Our investigation of AKAP6 and PLN mutants (R9C, L39Stop, RΔ14, and S16A) provides some insights; for example, we detected a higher level of PLN‐R9C phosphorylation upon isoproterenol activation which is consistent with the earlier proposed PKA “trapping” by the PLN‐R9C structure (Schmitt et al. 2003). Furthermore, PLN‐L39Stop showed a significantly higher level of phosphorylation upon isoproterenol treatment. This result might be due to misrouted localization of PLN‐L39Stop to other subcellular regions and its interaction with different kinase populations in the cytosol as well as membranous compartments. Similarly, we detected a higher level of AKAP6 pull‐down with PLN (S16A) compared to PLN (WT) both with and without adrenergic stimulation. Based on our phospho‐PLN immunoblots and in accordance with previous literature, it is known that PLN (S16A) cannot be phosphorylated, and therefore, it is possible that a more stable AKAP6‐S16A complex forms within the precipitated protein. Finally, our study showed lack of PLN phosphorylation in PLN‐RΔ14. This observation might be due to the distortion in PKA recognition site (R‐R‐X‐S) because of the lack of Arg14 in PLN structure (Masterson et al. 2010).

In conclusion, our study showed high colocalization and biochemical interaction between AKAP6 and PLN in HEK‐293T. This finding was further corroborated in rat cardiac tissues and isolated adult cardiomyocytes. In adult cardiomyocytes, the regulation of protein phosphorylation events by different PKA pool is highly compartmentalized. Although several unknown questions about the Ca^2+^ signaling events at the nuclear level still remain unanswered, our findings showed that the regulation of Ca^2+^ signaling proteins such as PLN is highly specialized and was dependent upon their spatial localization and the regulators that exist in their proximity. Consistently, Chen et al. (2018) showed that perinuclear PLN of mouse ventricular myocytes modulated nuclear Ca^2+^ handling by controlling RyR2 and IP3R activities. Given that the specificity of AKAP6 is due to its precise localization to the nuclear region in mature myocytes, our findings support a critical role of AKAP6 in regulating phosphorylation of several important nuclear Ca^2+^‐cycling proteins, with clear potential to affect critical transcriptional events downstream. Future studies examining and clarifying its impact are required to delineate its role more fully.

## Conflict of Interest

The authors declare that they have no conflict of interests with the contents of this article.

## Supporting information




**Figure S1.** Full blots of protein expression of AKAP6.
**Figure S2.** Full blots of AKAP6 and PLN immunoprecipitation changes upon mutation and adrenergic stimulation.
**Figure S3.** Column and scatterplot analysis for AKAP6 and PLN immunoprecipitation changes upon mutation and adrenergic stimulation.
**Table S1.** List of the main membranous AKAPs found in the human heart.
**Table S2.** Normalized Mean and SEM of RNA‐seq data for AKAPs in human hearts.
**Table S3.** Normalized Mean and SEM of RNA‐seq data of human AKAP candidates in non‐hypertrophic and hypertrophic adult human.
