# Supplementary material for: AKAP6 and phospholamban colocalize and interact in HEK‐293T cells and primary murine cardiomyocytes
Source: Physiol Rep. 2019 Jul 19;7(14):e14144. doi: 10.14814/phy2.14144 (PMC6642276; doi:10.14814/phy2.14144)
Supplement: Supplementary file 1 — Figure S1. Full blots of protein expression of AKAP6. Figure S2. Full blots of AKAP6 and PLN immunoprecipitation changes upon mutation and adrenergic stimulation. Figure S3. Column and scatterplot analysis for AKAP6 and PLN immunoprecipitation changes upon mutation and adrenergic stimulation. Table S1. List of the main membranous AKAPs found in the human heart. Table S2. Normalized Mean and SEM of RNA‐seq data for AKAPs in human hearts. Table S3. Normalized Mean and SEM of RNA‐seq data of human AKAP candidates in non‐hypertrophic and hypertrophic adult human. [file PHY2-7-e14144-s001.pdf]

AKAP6 and phospholamban Co-localize and Interact in HEK-293T cells and primary murine cardiomyocytes

Farigol Hakem Zadeh [1,2], Allen C. T. Teng [1,2], Uros Kuzmanov [2], Paige J. Chambers [3],

A. Russell Tupling [3], Anthony O. Gramolini[1,2]*

This file includes:
Supporting information: Material and Methods

Immunoblotting

Protein samples were extracted from the HEK-293T cells (IP solution), CMNCs (RIPA solution) and heart (RIPA solution) as described in the material and methods section. The supernatants containing soluble proteins were kept. Using the Bradford reagent (Sigma) the concentration of each protein lysate was determined. The lysates were then subjected to Sodium Dodecyl Sulfate-Polyacrylamide Gel Electrophoresis. After the transfer, the membrane was blocked in blocking solution (5% skim milk, 0.05% Tris solution saline-Tween 20 (TBS-T)) for 1 hr at room temperature. The resulted bands were compared in adjusted volumes using the volume tools of ImageLab Software (version 5.2.1).

Immunofluorescence of HEK293-T

Cells were in phosphate-buffered saline (PBS), fixed with 4% paraformaldehyde on ice and permeabilized with ice cold 90% methanol at -20°C for 1 min. After permeabilization, the cells were blocked with blocking solution (5% FBS in PBS) at room temperature for 1 hr. The cells were incubated overnight at 4°C with the primary antibodies diluted in blocking solution. The next day, the cells were washed in PBS. The fluorophore-conjugated secondary antibodies were diluted in blocking solution, and the staining of the cells was performed at room temperature for 1 hr in the dark. The samples were counterstained with 4', 6-Diamidino-2- Phenylindole (DAPI, Sigma-Aldrich) or Hoechst 33342 (nuclear stain, Sigma) for 30 min in the dark. The slides were mounted in the Fluoromount medium (Sigma). The slides were visualized and Z-stacked with a Zeiss spinning disk confocal microscopy (Zeiss Observer.Z1). A three-dimensional interpretation of the multichannel Z-stack images was performed using Imaris software.

Immunofluorescence of Neonatal and Adult Ventricular Myocytes

Isolated cells were washed gently with PBS at 4°C for 20 min. The cells were then fixed with 4% PFA for 30 min at 4°C. Then, they were incubated at 4°C with fresh permeabilization solution (0.2% Tween-20, 0.5% Triton X-100 in 1x PBS). The cells were then washed gently with PBS at 4°C three times for 5 min each. The cells were then incubated in blocking solution (5% FBS, 0.2% Tween-20, 0.5% Triton X-100 in PBS) for 30 min at room temperature. They were then incubated overnight at 4°C with primary antibody which was diluted in blocking solution with gentle shaking. The primary antibodies used for these experiments were mouse monoclonal 2D12 anti-PLN antibody (Abcam, 1:500) and mouse anti-AKAP6 antibody (OR017.720 - Biolegend, 1:500). The next day, the cells were gently washed with the permeabilization solution twice (15 min each) with PBS. The fluorescent conjugated secondary antibodies (Alexa Fluor 488, 1:500 or 633, 1:500, Life Technologies) diluted in blocking solution were applied to the cells and then incubated in the dark for 1 hr at room temperature. Afterwards, the secondary antibodies were removed, and the cells were washed twice (15-min each) with PBS in the dark at room temperature. The cells were then counterstained with 4', 6-Diamidino-2- Phenylindole (DAPI, Sigma-Aldrich) or Hoechst 33342 (nuclear stain, Sigma) for 30 min in the dark. Subsequently, the cells were washed twice (15 min each) with PBS in the dark. The cells were

then visualized and Z-stacked using. A three-dimensional interpretation of the multichannel Z-stack images was performed using Imaris software (Bitplane).

Transduction of CMNC

Polybrene was diluted to the final concentration of 10 µg/mL in Prewarmed DMEM/F12. The amount of this mixture was the same as the amount of virus required (2 mL per each well of a 6-well plate). The mixture was then gently administered on the CMNCs and incubated at 37°C for 90 min. During this time, the required amount of viral stock was thawed at room temperature. After the incubation time, the mixture was removed, and the virus was added to the culture. CMNCs were then incubated for 21 hr with lentiviral solution at 37°C, 5% CO_2. The next day the media was changed to DMEM/F12 supplemented with 2% FBS and 1% P/S for an additional 24 hr prior to any downstream application. For the cells were kept for 96 hr post-transduction while the media was changed every other day.

SERCA1 activity measurements

Cells were collected and homogenized in phenylmethylsulfonyl fluoride (PMSF) solution (250mM sucrose, 5nM HEPES, 0.2mM phenylmethylsulfonyl fluoride, 0.2% sodium azide, and 1%triton X-100), sonicated (2secs on: 5 secs off for 20secs total). They were then centrifuged at 10,000g for 30 min at 4°C. The supernatant (75ul) was then diluted into 5ml of ATPase assay solution (100 mM KCl, 20 mM HEPES, 10 mM $MgCl_2$, 10 mM NaN_3, 10 mM phosphoenolpyruvate, 5 mM ATP, 1 mM EGTA, pH 7.0), 18U/ml lactate dehydrogenase, 18 U/ml pyruvate kinase, and 4uM ionophore A23187 (Sigma C7522). Each reaction was measured in duplicate with a reaction volume of 100 µl. The reaction was started by adding 0.3 mM NADH. Total ATPase activity was measured using a spectrophotometric plate reader (SPECTRAMAX plus; Molecular Devices) across Ca^{2+} concentrations ranging from *p*Ca 7.0 to 4.5. Basal activity was determined in the presence of 40 µM of the Ca^{2+}-ATPase inhibitor cyclopiazonic acid in dimethyl sulfoxide. The free Ca^{2+} concentration corresponding to each $CaCl_2$ addition was assessed separately using dual-emission spectrofluorometry and the fluorescent Ca^{2+}-binding dye indo 1. The data were analyzed by nonlinear regression with computer software (Graph Pad Software), and the apparent Ca^{2+} affinity (*KCa*) values were calculated by using an equation for a general cooperative model for substrate activation. The values for maximal SERCA2a activity that occurred at *pCa* 6.423 to 5.407 were taken directly from the experimental data and normalized to protein content measured by the BCA assay.

Adenoviral transduction of AVC

The adenoviruses used in this experiment were kindly contributed by Kapiloff laboratory. The cardiomyocytes were transfected with a titer of 1000 MOI for 72 hr with Ad-U6 AKAP6 7210D mp2A adenovirus containing rat specific AKAP6 knock down shRNA and rAKAP6 7210D mut.C adenovirus containing control scrambled shRNA. The media was collected after 72 hr and the cells were fixed for IF.

Imaris analysis

The 3D reconstructions and colocalization analysis were performed using imaris software and automatic thresholding developed by Costes and Lockett at the National Institute of Health, NCI/SAIC was used (Costes et al., 2004).

Statistical analysis

Using GraphPad Prism 5, one-way ANOVA analysis was utilized to test the differences and the significance among more than two groups. To compared two groups unpaired t-test was performed. The data are expressed as mean ± SEM. A P-value of < 0.05 was considered significant.

Supporting information: Figures

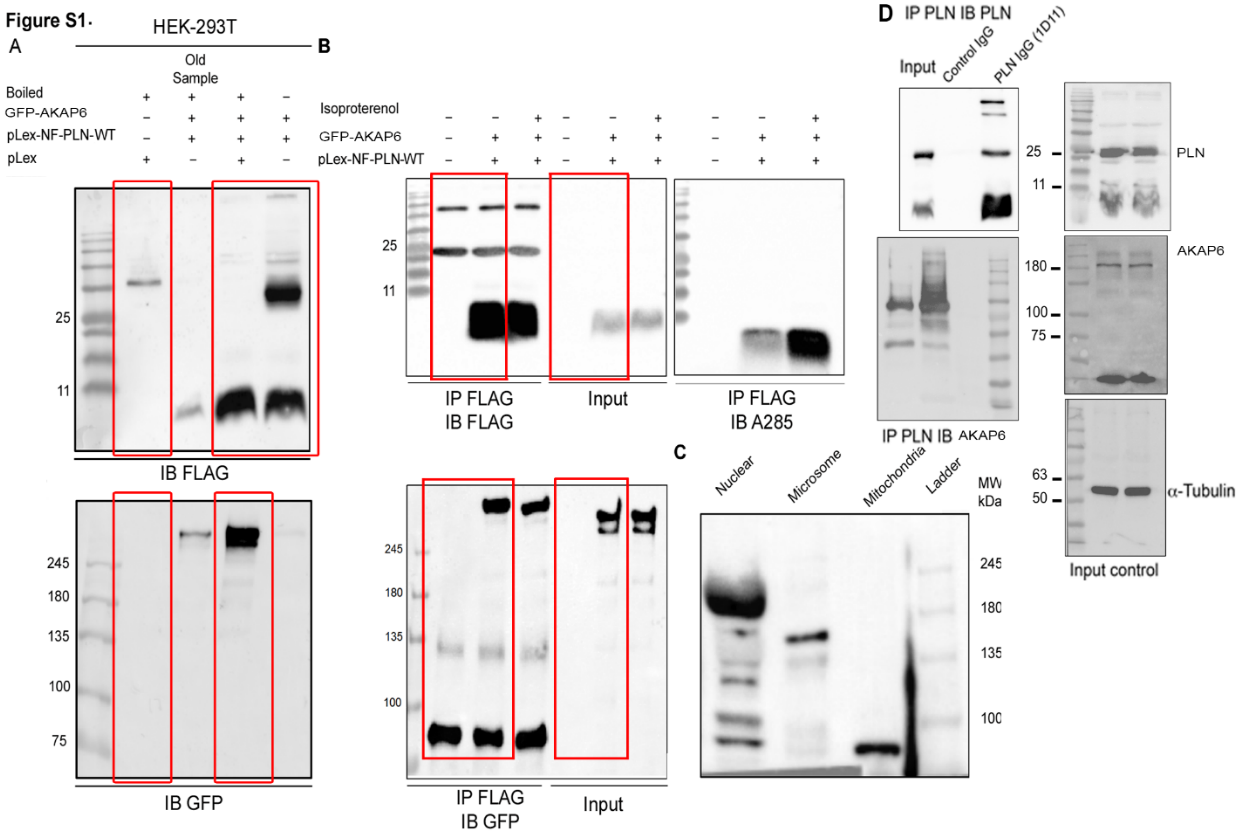

Figure S1. **Full blots of protein expression of AKAP6.** (A) Endogenous AKAP6 expression in CMNCs and adult heart at ~245 kDa, GFP-AKAP6 overexpression in HEK293-T cells at ~300 kDa. (B) Confirmation of overexpression of GFP-AKAP6 and NF-PLN-WT in pEGFPN1- AKAP6 and pLex-NF-PLN-WT co-transfected HE-293-T cells. (C) AKAP6 subcellular localization in adult heart. (D) IP of PLN with AKAP6 with and without isoproterenol treatment.

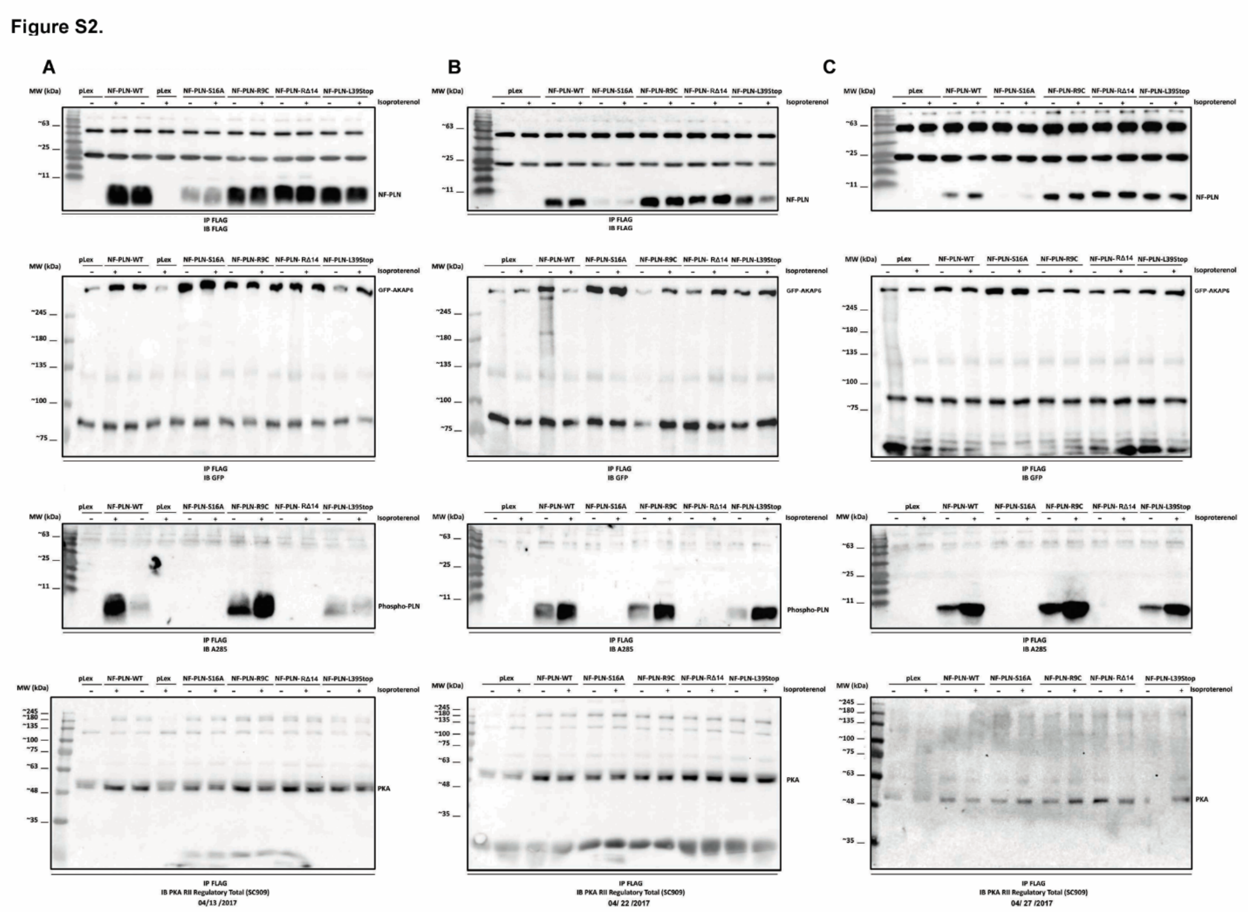

Figure S2. Full blots of AKAP6 and PLN immunoprecipitation changes upon mutation and adrenergic stimulation. IP was performed with Flag PLN. PLN, phoshoPLN, GFP-AKAP6 and PKA were detected for all mutants. (A) Blot1, (B) Blot2, (C) Blot3.

Figure S3.

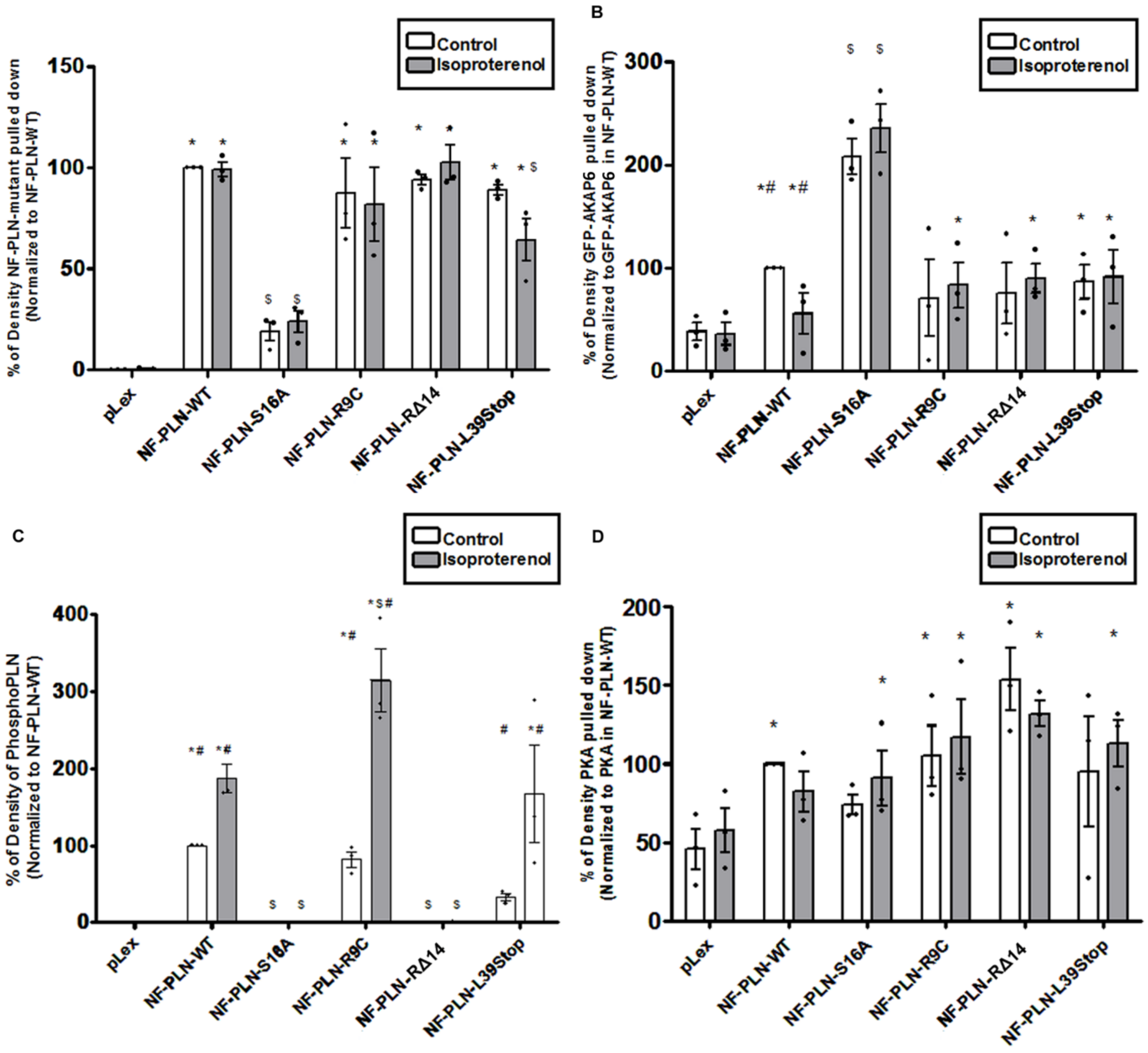

Figure S3. Column and scatterplot analysis for AKAP6 and PLN immunoprecipitation changes upon mutation and adrenergic stimulation. IP was performed with Flag PLN. PLN, phoshoPLN, GFP-AKAP6 and PKA were detected for all mutants. (A) Normalized Flag-PLN. (B) Normalized percentage of GFP-AKAP6. (C) Relative percentage of GFP-AKAP6. (D) Normalized percentage Phospho-PLN and (E) Relative percentage of phosphor-PLN. (F) Normalized percentage PKA. (E) Relative percentage of PKA pull down. These results are expressed as mean ± SEM; n=3. * p<0.05 versus pLex. $^{\$}$p<0.05 versus PLN-WT. $^{\#}$p<0.05, control vs. isoproterenol treatment.

REFERENCE

COSTES, S. V., DAELEMANS, D., CHO, E. H., DOBBIN, Z., PAVLAKIS, G. & LOCKETT, S. 2004. Automatic and quantitative measurement of protein-protein colocalization in live cells. *Biophys J,* 86**,** 3993-4003.

Table S1. List of the main membranous AKAPs found in the human heart.

Name	Aliases/Other names
AKAP1	A-Kinase Anchoring Protein 1; Dual Specificity A-Kinase-Anchoring Protein 1; Protein Phosphatase 1, Regulatory Subunit 43; A-Kinase Anchor Protein 1, Mitochondrial; Spermatid A-Kinase Anchor Protein 84; A Kinase (PRKA) Anchor Protein 1; A-Kinase Anchor Protein 149 KDa; Tudor Domain Containing 17; Dual-Specificity A-Kinase Anchoring Protein 1; Protein Kinase A Anchoring Protein 1; Protein Kinase A-Anchoring Protein 1; Protein Kinase Anchoring Protein 1; Testicular Secretory Protein Li 5; Protein Kinase A1; AKAP 149; D-AKAP-1; AKAP149; PRKA1; S-AKAP84; AKAP121; D-AKAP1; PPP1R43; SAKAP84; AKAP84; TDRD17; AKAP
AKAP2	A-Kinase Anchoring Protein 2; A Kinase (PRKA) Anchor Protein 2; A-Kinase Anchor Protein 2; Protein Kinase A2; AKAP-2; PRKA2; Protein Kinase A Anchoring Protein 2; Protein Kinase A-Anchoring Protein 2, KIAA0920; AKAP-KL; AKAPKL; MISP2;
PALM2-AKAP2	PALM2-AKAP2 Readthrough; PALM2-AKAP2 Readthrough Transcript; PALM2-AKAP2 Protein; AKAP2
AKAP3	A-Kinase Anchoring Protein 3; Fibrous Sheath Protein Of 95 KDa; Cancer/Testis Antigen 82; Protein Kinase A-Anchoring Protein 3; A Kinase (PRKA) Anchor Protein 3; A-Kinase Anchor Protein 3; Fibrousheathin I; Fibrousheathin-1; AKAP 110; AKAP110; FSP95; PRKA3; CT82; SOB1; Epididymis Secretory Sperm Binding Protein; Protein Kinase A Binding Protein AKAP 110; A-Kinase Anchor Protein, 110kDa; 3A-Kinase Anchor Protein 110 KDa; Epididymis Luminal Protein 159; Sperm Oocyte-Binding Protein 1; Sperm Oocyte-Binding Protein; HEL159; AKAP-3
AKAP4	A-Kinase Anchoring Protein 4; A-Kinase Anchor Protein 82 KDa; Protein Kinase A Anchoring Protein 4; Major Sperm Fibrous Sheath Protein; A Kinase (PRKA) Anchor Protein 4; A-Kinase Anchor Protein 4; Cancer/Testis Antigen 99; Testis-Specific Gene HI; AKAP 82; HAKAP82; AKAP-4; AKAP82; PRKA4; HI; Epididymis Secretory Sperm Binding Protein; Protein Kinase A-Anchoring Protein 4; CT99; FSC1; P82
AKAP5	A-Kinase Anchoring Protein 5; CAMP-Dependent Protein Kinase Regulatory Subunit II; High Affinity-Binding Protein; A Kinase (PRKA) Anchor Protein 5; A-Kinase Anchor Protein 79 KDa; A-Kinase Anchor Protein 5; AKAP 79; AKAP79; H21; A-Kinase Anchoring Protein 75/79; AKAP75; AKAP-5
AKAP6	A-Kinase Anchoring Protein 6; Protein Kinase A Anchoring Protein 6; A Kinase (PRKA) Anchor Protein 6; A-Kinase Anchor Protein 100 KDa; A-Kinase Anchor Protein 6; AKAP 100; AKAP100; AKAP-6; PRKA6; MAKAP; Protein Kinase A-Anchoring Protein 6; Muscle A-Kinase Anchoring Protein; KIAA0311; ADAP100; ADAP6
AKAP7	A-Kinase Anchoring Protein 7; A Kinase (PRKA) Anchor Protein 7; A-Kinase Anchor Protein 18 KDa 3; AKAP 18; AKAP15; AKAP18; Protein Kinase A-Anchoring Protein 7 Isoforms Alpha/Beta; Protein Kinase A-Anchoring Protein 7 Isoform Gamma 4; A-Kinase Anchor Protein 7 Isoforms Alpha And Beta; A-Kinase Anchor Protein 7 Isoform Gamma; AKAP-7 Isoforms Alpha And Beta; A-Kinase Anchor Protein 9 KDa; PRKA7 Isoforms Alpha/Beta; AKAP-7 Isoform Gamma; PRKA7 Isoform Gamma
AKAP8	A-Kinase Anchoring Protein 8; A Kinase (PRKA) Anchor Protein 8; A-Kinase Anchor Protein, 95kDa; A-Kinase Anchor Protein 8; AKAP 95; AKAP-8; AKAP95; A-Kinase Anchor Protein 95 KDa; AKAP-95
AKAP8L	A-Kinase Anchoring Protein 8 Like; A Kinase (PRKA) Anchor Protein 8-Like; A-Kinase Anchor Protein 8-Like; Homologous To AKAP95 Protein; Neighbor Of AKAP95; AKAP8-Like Protein; NAKAP95; HAP95; NAKAP; HA95; Testis Tissue Sperm-Binding Protein Li 90mP; Neighbor Of A-Kinase Anchoring Protein 95; Neighbor Of A-Kinase-Anchoring Protein 95; Neighbor Of A Kinase Anchoring Protein 95; Helicase A-Binding Protein 95 KDa; Helicase A-Binding Protein 95 4
AKAP9	A-Kinase Anchoring Protein 9; Centrosome- And Golgi-Localized Protein Kinase N-Associated Protein; Centrosome- And Golgi-Localized PKN-Associated Protein; Protein Phosphatase 1, Regulatory Subunit 45; Protein Kinase A Anchoring Protein 9; A Kinase (PRKA) Anchor Protein 9; A-Kinase Anchor Protein 350 KDa; A-Kinase Anchor Protein 450 KDa; A-Kinase Anchor Protein 9; AKAP9-BRAF Fusion Protein; AKAP 120-Like Protein; Protein Hyperion; Protein Yotiao; AKAP350; AKAP450; AKAP-9; CG-NAP; PRKA9; A Kinase (PRKA) Anchor Protein (Yotiao) 9; Protein Kinase A-Anchoring Protein 9; A-Kinase Anchor Protein, 350kDa; A-Kinase Anchoring Protein 450; Kinase N-Associated Protein; AKAP120-Like Protein; MU-RMS-40.16A; HgAKAP 350; HYPERION; AKAP 350; AKAP 450; KIAA0803; PPP1R45; YOTIAO; Yotiao; LQT11 3
AKAP10	A-Kinase Anchoring Protein 10; Dual Specificity A Kinase-Anchoring Protein 2; Mitochondrial A Kinase PPKA Anchor Protein 10; A-Kinase Anchor Protein 10, Mitochondrial; Protein Kinase A Anchoring Protein 10; A Kinase (PRKA) Anchor Protein 10; D-AKAP-2; AKAP-10; PRKA10; Dual-Specificity A-Kinase Anchoring Protein 2; Protein Kinase A-Anchoring Protein 10; A Kinase Anchor Protein 10; D-AKAP2 3
AKAP11	A-Kinase Anchoring Protein 11; Protein Phosphatase 1, Regulatory Subunit 44; Protein Kinase A Anchoring Protein 11; A-Kinase Anchoring Protein, 220kDa; A Kinase (PRKA) Anchor Protein 11; A-Kinase Anchor Protein 220 KDa; A-Kinase Anchor Protein 11; AKAP 220; AKAP-11; AKAP220; PRKA11; Protein Kinase A-Anchoring Protein 11; A Kinase Anchor Protein 220 KDa; KIAA0629; HAKAP220; PPP1R44
AKAP12	A-Kinase Anchoring Protein 12; A Kinase (PRKA) Anchor Protein 12; Src-Suppressed C Kinase Substrate; A-Kinase Anchor Protein 12; AKAP 250; AKAP250; Gravin; A Kinase (PRKA) Anchor Protein (Gravin) 12; Myasthenia Gravis Autoantigen Gravin; A-Kinase Anchor Protein, 250kDa; A-Kinase Anchor Protein 250 KDa; Kinase Scaffold Protein Gravin; Myasthenia Gravis Autoantigen; AKAP-12; SSeCKS
AKAP13	A-Kinase Anchoring Protein 13; Lymphoid Blast Crisis Oncogene; Breast Cancer Nuclear Receptor-Binding Auxiliary Protein; Non-Oncogenic Rho GTPase-Specific GTP Exchange Factor; Guanine Nucleotide Exchange Factor Lbc; Protein Kinase A-Anchoring Protein 13; Human Thyroid-Anchoring Protein 31; A Kinase (PRKA) Anchor Protein 13; A-Kinase Anchor Protein 13; LBC Oncogene; AKAP-Lbc; AKAP-13; PRKA13; LBC; BRX; P47; PROTO-LBC; ARHGEF13; PROTO-LB; C-Lbc; HA-3; Ht31; HT31 4
AKAP14	A-Kinase Anchoring Protein 14; Protein Kinase A-Anchoring Protein 14; A Kinase (PRKA) Anchor Protein 14; A-Kinase Anchor Protein 28 KDa; A-Kinase Anchor Protein 14; AKAP 28; AKAP-14; AKAP28; PRKA14; A-Kinase Anchoring Protein 28
AKAP17A	A-Kinase Anchoring Protein 17A; Splicing Factor, Arginine/Serine-Rich 17A; Protein Kinase A-Anchoring Protein 17A; A Kinase (PRKA) Anchor Protein 17A; A-Kinase Anchor Protein 17A; AKAP-17A; DXYS155E; CXYorf3; SFRS17A; PRKA17A; 721P; XE7; Chromosome X And Y Open Reading Frame 3; B-Lymphocyte Surface Antigen; Pseudoautosomal Gene XE7; B-Lymphocyte Antigen; Protein XE7; CCDC133; XE7Y
SYNM	Synemin; Desmuslin; Synemin, Intermediate Filament Protein; Synemin Alpha; Synemin Beta; DMN; SYN; EC 2.6.1.16; EC 1.4.99.1; KIAA0353
C2orf88	Chromosome 2 Open Reading Frame 88; Small Membrane AKAP; Small Membrane A-Kinase Anchor Protein; SmAKAP; Small A-Kinase Anchoring Protein

Table S2. Normalized Mean and SEM of RNA-seq data for AKAPs in human hearts. HPM (n=85, individuals, unit: Fragments Per Kilobase Mil-lion (FPKM)), HPA (n=4, heart tissues, unit: Transcripts Per Kilobase Million (TPM)), GTEx (n=412, heart tissues, unit: Reads Per Kilobase Million(RPKM)), FANTOM5 (n=4, individuals, unit: Tags Per Million) and GEO (n=4, individuals, unit: Reads Per Kilobase Million(RPKM)) database. An 'X' is incorporated where RNA-seq data is not found. The data shows the Mean± SEM expressions normalized to the maximum mean signal all across within each dataset, except for the HPM results that only provided Mean expression values.

Dataset	AKAP1			AKAP2			PALM2-AKAP2			AKAP3		
	MEAN	SEM	n	MEAN	SEM	n	MEAN	SEM	n	MEAN	SEM	n
HPM (FPKM)	0.047703	n.a.	85	0.272435	n.a.	85	0.284309	n.a.	85	0	n.a.	85
HPA (TPM)	0.6402037	0.100196	4	0.4895147	0.05580139	4	0.2360695	0.01603992	4	0.00958658	0.00097843	4
GTEx (RPKM)	0.508478	0.01128645	412	0.5101357	0.01728191	412	X	X	X	0.02067651	0.00124634	412
FANTOM5 (Tag/million)	0.4482759	0.0583817	4	1	0.2167975	4	0.2208945	0.07272107	4	0.00648686	0.00034141	4
GEO (RPKM)	0.398237	0.04770199	4	0.0013712	0.00026118	4	0.00117532	7.689E-20	4	0.0072478	0.00182827	4

Dataset	AKAP4			AKAP5			AKAP6			AKAP7		
	MEAN	SEM	n	MEAN	SEM	n	MEAN	SEM	n	MEAN	SEM	n
HPM (FPKM)	0	n.a.	85	0	n.a.	85	0	n.a.	85	0	n.a.	85
HPA (TPM)	0	0	4	0.0014979	0.00029958	4	0.431396	0.05043642	4	0.05032954	0.00605123	4
GTEx (RPKM)	0	0	412	0.0005785	4.97854E-05	412	0.1505386	0.004446261	412	0.02581815	0.000536231	412
FANTOM5 (Tag/million)	0	0	4	0.0006828	0.00068283	4	0.2574257	0.02663025	4	0.03618983	0.00887675	4
GEO (RPKM)	0	0	4	0.0041136	0.00052236	4	0.4133203	0.03336285	4	0.03506366	0.00882238	4

Dataset	AKAP8			AKAP8L			AKAP9			AKAP10		
	MEAN	SEM	n	MEAN	SEM	n	MEAN	SEM	n	MEAN	SEM	n
HPM (FPKM)	0	n.a.	85	0	n.a.	85	0.023885	n.a.	85	0	n.a.	85
HPA (TPM)	0.0386459	0.00674999	4	0.1348113	0.01797816	4	0.05721989	0.00583734	4	0.05632115	0.00520041	4
GTEx (RPKM)	0.1202238	0.001618367	412	0.3586735	0.005465889	412	0.05608435	0.000985082	412	0.05453361	0.000818243	412
FANTOM5 (Tag/million)	0.1013998	0.05360191	4	0.2355753	0.00204848	4	0.3048822	0.1048139	4	0.1433937	0.05735746	4
GEO (RPKM)	0.04936337	0.00296646	4	0.1571009	0.00171268	4	0.1402546	0.0331886	4	0.03976494	0.00431543	4

Dataset	AKAP11			AKAP12			AKAP13			AKAP14		
	MEAN	SEM	n	MEAN	SEM	n	MEAN	SEM	n	MEAN	SEM	n
HPM (FPKM)	0.196069	n.a.	85	0.045403	n.a.	85	0.030974	n.a.	85	0	n.a.	85
HPA (TPM)	0.09017376	0.01472828	4	0.1315159	0.03547174	4	0.5029958	0.08134978	4	0	0	4
GTEx (RPKM)	0.07008478	0.001431403	412	0.2691728	0.01604918	412	0.2815982	0.005859933	412	4.86126E-06	4.86126E-06	412
FANTOM5 (Tag/million)	0.0938887	0.02833732	4	0.3188802	0.1017412	4	0.4127689	0.02970297	4	0	0	4
GEO (RPKM)	0.06483839	0.00276409	4	0.0532811	0.0094928	4	0.3089128	0.0248864	4	0	0	4

Dataset	AKAP17A			SYNM			C2orf88		
	MEAN	SEM	n	MEAN	SEM	n	MEAN	SEM	n
HPM (FPKM)	0.030932	n.a.	85	1	n.a.	85	0.033673	n.a.	85
HPA (TPM)	0.08867585	0.01052661	4	1	0.1834546	4	0.09077292	0.00871534	4
GTEx (RPKM)	0.2103418	0.003936192	412	1	0.02365018	412	0.05245299	0.001118255	412
FANTOM5 (Tag/million)	0.01877774	0.01877774	4	0.5421646	0.1454421	4	0.1160806	0.04028679	4
GEO (RPKM)	0.05504407	0.00370289	4	1	0.1170628	4	0.09265426	0.01357392	4

Table S3. Normalized Mean and SEM of RNA-seq data **of human AKAP candidates in non-hypertrophic and hypertrophic adult human**. RNA seq data were extracted from GEO data and was normalized relative to the highest expression across all samples. These results are shown as mean ± SEM; n=4 for normal and n=5 for hypertrophic samples. Two-tailed unpaired t-test. *p<0.05 comparing normal versus hypertrophic patients' RNA expression in the heart for each AKAP.

	Non-hypertrophic				Hypertrophic					p-values (*p<0.05)
	S1	S2	S3	S4	S1	S2	S3	S4	S5	
AKAP1	0.300882	0.459941	0.419196	0.412928	0.270323	0.338491	0.279725	0.308717	0.288344	0.0181
AKAP2	0.001567	0.001567	0.000784	0.001567	0.000784	0	0	0.001567	0.001567	0.2168
PALM2-AKAP2	0.000784	0.000784	0.000784	0.002351	0.001567	0.002351	0.001567	0.003918	0.002351	0.0892
AKAP3	0.003918	0.010186	0.007835	0.007052	0.003918	0.007835	0.002351	0.002351	0.003918	0.0891
AKAP5	0.004701	0.004701	0.003134	0.003918	0.004701	0.005485	0.004701	0.003918	0.004701	0.2168
AKAP6	0.33379	0.383154	0.448972	0.487366	0.33379	0.299315	0.264838	0.328306	0.282861	0.0126
AKAP7	0.030558	0.054848	0.026641	0.028208	0.020372	0.040744	0.027424	0.017238	0.016454	0.2134
AKAP8	0.057982	0.054065	0.047796	0.03761	0.058766	0.065034	0.058766	0.051714	0.054065	0.1198
AKAP8L	0.152791	0.157492	0.152007	0.166112	0.14809	0.152007	0.157492	0.130069	0.15044	0.1576
AKAP9	0.083839	0.065818	0.173164	0.238198	0.0619	0.047796	0.091675	0.056415	0.073653	0.0812
AKAP10	0.041528	0.047796	0.032909	0.036827	0.045446	0.04858	0.054848	0.043095	0.0619	0.0548
AKAP11	0.055632	0.058766	0.065034	0.079922	0.089324	0.076004	0.084623	0.065034	0.093242	0.0582
AKAP12	0.05093	0.07522	0.043879	0.043095	0.12145	0.082272	0.194319	0.069736	0.188051	0.0364
AKAP13	0.262488	0.329089	0.343193	0.300882	0.416847	0.362782	0.343193	0.532811	0.397257	0.0412
AKAP14	0	0	0	0.000001	0	0.001567	0	0	0	0.4074
AKAP17A	0.062684	0.050147	0.054065	0.053281	0.0619	0.058766	0.067385	0.060333	0.065818	0.0354
SYNM	0.741235	0.855631	1.135358	1.267777	1.004506	1.017042	1.024878	1.283448	1.289716	0.375
C2orf88	0.068168	0.090891	0.115181	0.096376	0.068168	0.068168	0.061117	0.050147	0.049363	0.011
